# Transcriptional dynamics orchestrating the development and integration of neurons born in the adult hippocampus

**DOI:** 10.1126/sciadv.adp6039

**Published:** 2024-07-19

**Authors:** Natalí B. Rasetto, Damiana Giacomini, Ariel A. Berardino, Tomás Vega Waichman, Maximiliano S. Beckel, Daniela J. Di Bella, Juliana Brown, M. Georgina Davies-Sala, Chiara Gerhardinger, Dieter Chichung Lie, Paola Arlotta, Ariel Chernomoretz, Alejandro F. Schinder

**Affiliations:** ^1^ Instituto de Investigaciones Biomédicas de Buenos Aires (IIBBA) – CONICET, Buenos Aires, Argentina.; ^2^ Laboratory of Neuronal Plasticity, Leloir Institute, Buenos Aires, Argentina.; ^3^ Laboratory of Integrative Systems Biology, Leloir Institute, Buenos Aires, Argentina.; ^4^ Department of Stem Cells and Regenerative Biology, Harvard University and Stanley Center for Psychiatric Research, Broad Institute of MIT and Harvard, Cambridge, MA, USA.; ^5^ Institute of Biochemistry, Friedrich-Alexander Universität Erlangen-Nürnberg, Erlangen, Germany.; ^ 6^ University of Buenos Aires, School of Science, Phys Dept and INFINA (CONICET-UBA), Buenos Aires, Argentina.

## Abstract

The adult hippocampus generates new granule cells (aGCs) with functional capabilities that convey unique forms of plasticity to the preexisting circuits. While early differentiation of adult radial glia-like cells (RGLs) has been studied extensively, the molecular mechanisms guiding the maturation of postmitotic neurons remain unknown. Here, we used a precise birthdating strategy to study aGC differentiation using single-nuclei RNA sequencing. Transcriptional profiling revealed a continuous trajectory from RGLs to mature aGCs, with multiple immature stages bearing increasing levels of effector genes supporting growth, excitability, and synaptogenesis. Analysis of differential gene expression, pseudo-time trajectory, and transcription factors (TFs) revealed critical transitions defining four cellular states: quiescent RGLs, proliferative progenitors, immature aGCs, and mature aGCs. Becoming mature aGCs involved a transcriptional switch that shuts down pathways promoting cell growth, such SoxC TFs, to activate programs that likely control neuronal homeostasis. aGCs overexpressing *Sox4* or *Sox11* remained immature. Our results unveil precise molecular mechanisms driving adult RGLs through the pathway of neuronal differentiation.

## Teaser

Specific newborn cell molecular profiling reveals critical transitions toward neuronal maturation with precise time resolution.

## INTRODUCTION

The hippocampus is involved in processing spatial representations and plays multiple functions in regard to memory acquisition, storage, and retrieval (*1*, *2*). The dentate gyrus is the primary gateway for information coming from the entorhinal cortex to the hippocampus. It bears an architecture with unique dynamics due to the presence of radial glia-like cells (RGLs), which continuously generate new neurons that integrate in the preexisting networks (*3*–*5*). Adult-born granule cells (aGCs) provide a substrate for the plasticity of perforant-path to granule cell (GC) synapses (dentate input) as well as for mossy fiber to CA3 connections (output) (*6*–*9*). Such intensive circuit remodeling is crucial for the fine discrimination of similar experiences (*10*–*12*).

**Fig. 1.**
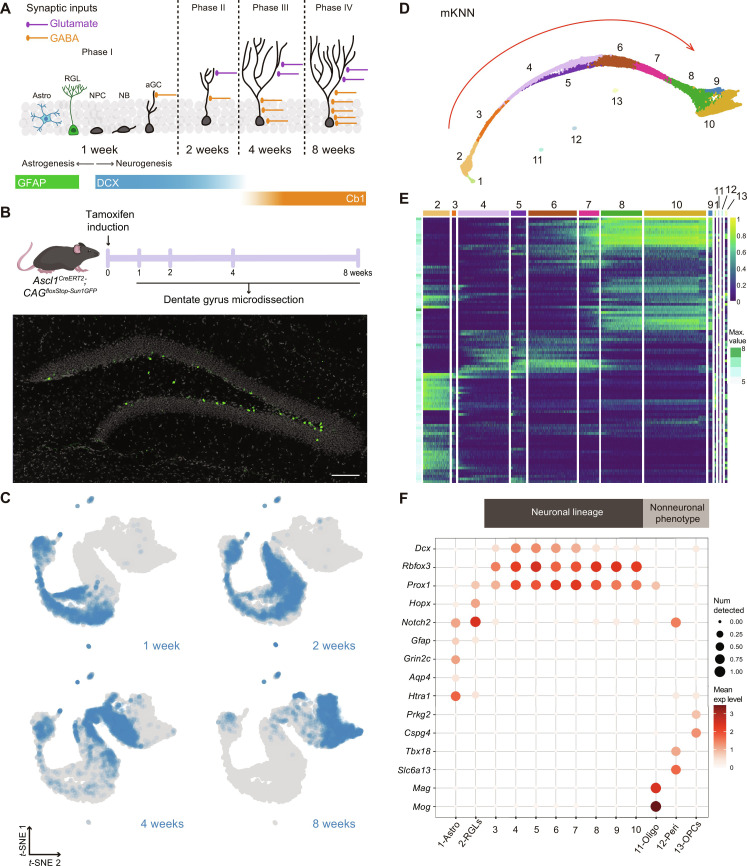
Transcriptional dynamics of neuronal cohorts born in the adult dentate gyrus. (**A**) Scheme of new neurons development in the adult hippocampus. Phase I: Radial glia-like cells (RGLs) generate astrocytes (Astro) or neural progenitor cells (NPCs). New granule cells (aGCs) receive depolarizing GABAergic inputs. Phase II: aGCs acquire glutamatergic contacts. Phase III: Period of enhanced excitation/inhibition balance. Phase IV: GABAergic inhibition matures and excitability decreases (*87*). Glial fibrillary acidic protein (GFAP) expression denotes astrocytic lineage. DCX and Calb1 denote neuronal lineage. (**B**) Experimental design. *Ascl1^CreERT2^;CAG ^floxStop-Sun1/sfGFP^* mice received tamoxifen (TAM) injections. Dentate gyri were microdissected at the indicated time points. Image depicts a dentate gyrus with Sun1/sfGFP-labeled nuclei from 2-week-old aGCs. Scale bar, 100 μm. (**C**) *t*-Distributed stochastic neighbor embedding (*t*-SNE) for individual cohorts collected 1, 2, 4, and 8 weeks after labeling. (**D**) Mutual *k*-nearest neighbor (mKNN; *k* = 40) graph displaying 13 clusters. Red arrow denotes the path followed by adult neurogenesis. (**E**) Heatmap showing the normalized expression for 100 genes with highest variability (table S1). Numbers and colors correspond to clusters in (D). Maximal expression is shown on the left (Max. value). Scale on the right denotes mean expression level. (**F**) Dot plot exhibiting canonical markers. Pericyte (Peri): *Tbx18* and *Slc6a13*; oligodendrocyte (Oligo): *Mag* and *Mog*; oligodendrocyte progenitor (OPC): *Prkg2* and *Cspg4*; astrocyte (Astro): *Grin2c*, *Aqp4*, and *Htra1*; RGL: *Gfap*, *Hopx*, and *Notch2*; neurons: *Dcx*, *Rbfox3*, and *Prox1*. Scales on the right correspond to log_2_(mean gene expression) and dot size for expressing fraction of nuclei. All data correspond to dataset 1.

In the mouse brain, developing aGCs display distinct functional characteristics until they become mature after >8 weeks, a time course that is known to last several months in primates (*3*, *13*). The maturation of functional properties over time involves reduction of membrane resistance, expression of voltage-gated channels, afferent and efferent synaptogenesis, and switch of γ-aminobutyric acid (GABA)–mediated signaling from excitation to inhibition (Fig. 1A). At 4 weeks, aGCs display a transient period of enhanced excitability and susceptibility to activity-dependent synaptic plasticity. This period represents a crucial contribution of neurogenesis to circuit remodeling and information processing in the dentate gyrus (*6*, *8*, *9*, *14*–*22*). Once mature, aGCs are functionally indistinguishable to GCs born during perinatal development (*23*, *24*). Each step of neuronal differentiation is precisely shaped by the activity and physiological conditions of local dentate networks. Behaviors that increase the dentate activity such as exercise, environmental enrichment, or spatial learning exert positive modulatory effects, while conditions that alter the adult neurogenic niche such as aging, inflammation, or neurodegeneration are typically detrimental for neurogenesis (*4*). The molecular mechanisms controlling the transitions throughout aGC development and their modulation remain unknown.

Recent studies have exploited the power of single-cell transcriptomics to provide comprehensive descriptions of the initial stages of adult neurogenesis, thus identifying RGLs with distinct potential for self-renewal and differentiation (*25*, *26*). Additional work has strengthened the long-standing notion that RGLs generate intermediate neural progenitor cells (NPCs) with high proliferating capacity, which then transition to postmitotic neuroblasts (*27*). Later steps of neuronal differentiation have been inferred after studying dentate gyrus cells isolated from postnatal and adult mice (*28*). However, the progression from RGL to mature neuron in the adult hippocampus has never been studied.

Here, we put forward the hypothesis that developmental transitions respond to distinctive molecular programs that are sequentially activated in aGCs. To capture all intermediate stages through the pathway of aGC differentiation, we labeled defined neuronal cohorts at different ages in vivo (from 1- to 8-week-old cells) and performed single-nucleus RNA sequencing (snRNA-seq), generating two independent datasets. The high specificity and temporal resolution of these datasets enabled us to reveal the profile of immature and mature aGCs. Unsupervised algorithms identified 10 clusters that assembled into a linear developmental trajectory. This continuous pathway was reflected in the transcriptional dynamics of effector genes including pathfinding and cell-adhesion molecules, ion channels, neurotransmitter receptors, and components of the synaptic machinery. Analysis of differential gene expression, pseudo-time trajectory, and transcription factors (TFs) revealed critical transitions defining four cellular states: quiescent neural stem cells, proliferative progenitors, postmitotic immature aGCs, and mature aGCs. The passage from immature to mature aGCs involved a transcriptional switch that shuts down TFs promoting cell growth and synaptogenesis and turned on the expression of pathways limiting growth and controlling homeostasis. The shutdown of *SoxC* genes emerged as a crucial event to achieve maturation. Supporting this hypothesis, overexpression of *Sox4* or *Sox11* maintained developing aGCs in a persistent immature phenotype. Overall, our work uncovers a molecular continuum underlying neuronal differentiation in the adult hippocampus, assigning specific transcriptomic profiles to the individual stages, revealing master regulator TFs and the effector molecules that convert those programs into neuronal function.

## RESULTS

### Transcriptomic profiling of adult neurogenesis with high temporal resolution

To profile neurogenesis in the adult mouse hippocampus, we used a strategy to isolate developing cells derived from RGLs expressing the proneural gene *Achaete-scute homolog 1* (*Ascl1*) (*25*, *29*, *30*). Young-adult *Ascl1^CreERT2^;CAG^floxStopSun1sfGFP^* mice were used to induce the expression of Sun1-sfGFP in the nuclear membrane of RGLs and permanently label their progeny upon tamoxifen (TAM) administration (Fig. 1B and fig. S1A) (*31*). This birthdating tag enabled a comprehensive analysis of the entire process encompassing neuronal differentiation and functional maturation. A first dataset was built using fluorescence-activated cell sorting (FACS)–sorted nuclei obtained from dentate gyri microdissected at 1, 2, 4, and 8 weeks after induction (cohorts w1 through w8). High-throughput *s*nRNA-seq carried out using Chromium 10x Genomics 3′ end sequencing technology rendered 14,367 profiles with a median of 2994 genes per nucleus belonging to all four cohorts (fig. S1, B to E). Plotting dataset 1 using *t*-distributed stochastic neighbor embedding (*t*-SNE) revealed that nuclei from the distinct cohorts formed a continuous sequence organized by neuronal age (Fig. 1C). Considering a mutual *k*-nearest neighbor graph (mKNN; *k* = 40), we used an unsupervised clustering procedure (Louvain) to group nuclei into communities (*32*). This initial unsupervised partition was further refined and revealed 13 clusters (Fig. 1D). Clusters #1 through #10 seemed to constitute a linear developmental trajectory, while clusters #11, #12, and #13 appeared separated from each other and distant from the main pathway. All clusters displayed individual signatures that supported the definition of the unsupervised partitions (Fig. 1E and table S1). The identity of each cluster was determined by the expression of canonical markers. The majority of cells (about 88% of dataset 1) were distributed in partitions #3 through #10 that belonged to the neuronal lineage and were identified by the expression *Rbfox3* (*NeuN*, panneuronal marker), *Prox1* (aGCs), and doublecortin (*Dcx*, immature neurons; Fig. 1F).

Because of their common ancestry, astrocytes and RGLs display similar transcriptional profiles that include *Gfap*, *Notch2*, and *HopX* (*33*). However, the comparison of differentially expressed genes (DEGs) revealed transcripts typically associated with astrocytic expression in cluster #1: *Glul* and *Htra1*. In addition, this analysis highlighted up-regulated RGL markers in cluster #2: *Sox5*, *Thrsp*, and *Lpar1* (tables S2 and S3). Moreover, the restricted expression of *Grin2c*, *Aqp4*, and *Htra1* identified cluster #1 as astrocytes and cluster #2 as RGLs (Fig. 1F) (*28*, *34*).

Clusters #11, 12, and 13 encompassed nonneuronal cells that included pericytes expressing *Tbx18* and *Slc6a13* (#11), oligodendrocytes expressing *Mag* and *Mog* (#12), and oligodendrocyte precursor cells expressing *Prkg2* and *Cspg4* (#13). Pericytes have not been reported as Ascl1 lineage, and their scarce representation in the entire dataset (~0.4%) suggests either a transient expression of Ascl1 in this population or a leaky expression of the CreERT2 recombinase. In contrast, oligodendrocyte progenitors were shown to activate Ascl1 (*35*). Therefore, the indelible labeling used here was expected to tag the oligodendrocytic progeny.

**Fig. 2.**
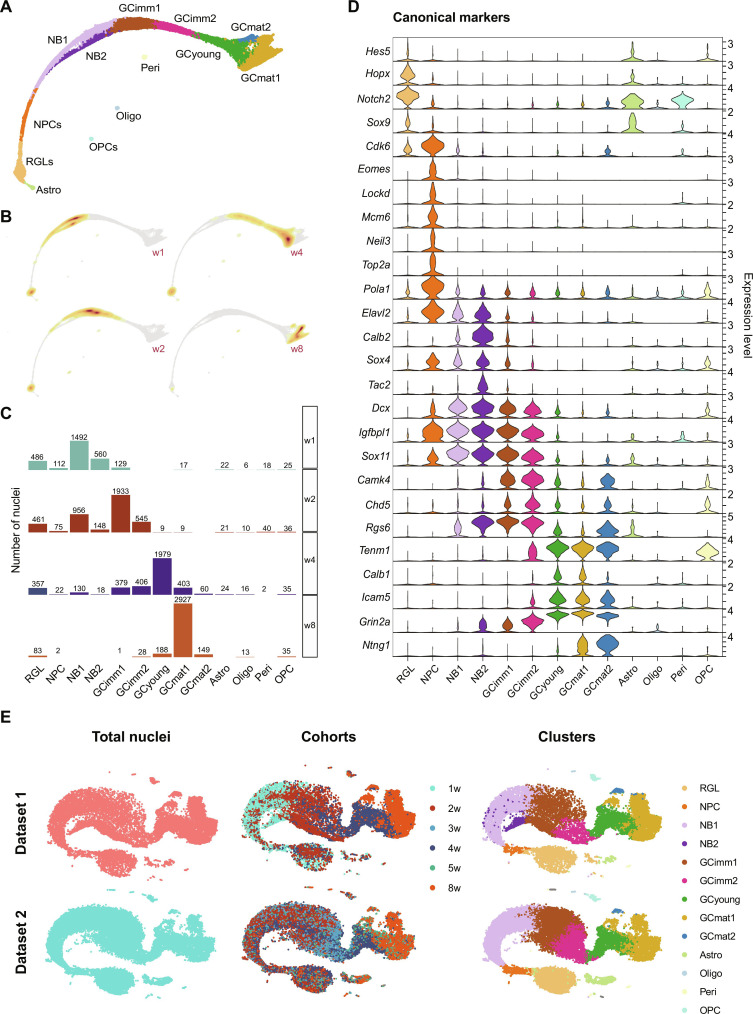
Timing and complexity of adult neurogenesis revealed by molecular profiling. (**A**) mKNN graph displaying cluster identity based on the expression of canonical genes: RGL, NPC, NB (neuroblast), GCimm (immature aGC), GCyoung (young aGC), GCmat (mature aGC), Astro, OPC, Oligo, and Peri. (**B**) Nuclei distribution for each cohort along clusters. (**C**) Progression of each cohort and their localization over the mKNN graph. Nuclei density is indicated by the yellow (low) to red (high) gradient. (**D**) Violin plots showing the expression level of canonical marker genes for the defined clusters. All data in the figure correspond to dataset 1. (**E**) *t*-SNE visualizations for the integration of datasets 1 and 2 (pink and turquoise, respectively, left panels), color-coded by cohort (middle panels) and by cluster (right panels).

Both the *t*-SNE and mKNN graphs indicated that aGCs follow a developmental continuum encompassing nine stages through neuronal maturation (Fig. 2A). While unsupervised clustering relied entirely on transcriptional profiles, nuclei followed a linear track ordered by neuronal age in the mKNN graphs (Fig. 2, B and C, and fig. S1, F and G). Clusters belonging to the neuronal lineage (*Rbfox3^+^* and *Prox1^+^*) followed a developmental sequence containing NPCs, neuroblasts (NB1 and NB2), immature neurons (GCimm1 and GCimm2), young neurons (GCyoung), and mature GCs (GCmat1 and GCmat2). NPCs were recognized by the expression of *Eomes*, *Top2a*, *Neil3*, *Cdk6*, *Lockd*, *Mcm6*, and *Pola1*, revealing cell cycle activity (Fig. 2D and fig. S5A). The presence of *Elavl2*, *Igfbpl1*, *Sox4*, and *Sox11* indicated that neuronal determination has already occurred in this early state. Neuroblasts lacked genes involved in cell cycle and expressed immature neuronal markers including *Calb2*, *Dcx*, *Rgs6*, *Sox4*, *Sox11*, and *Tac2*, constituting the first postmitotic stage of neuronal differentiation. Clusters that followed in the pathway, GCimm1 and GCimm2, shut down *Calb2* and *Elavl2* and expressed *Dcx*, *Sox11*, *Igfbpl1*, *Rgs6*, *Camk4*, and *Chd5*. GCyoung showed diminished expression of immature neuronal markers and enhanced levels of *Calb1*, *Icam5*, *Tenm1*, and *Grin2a.* Last, clusters belonging to mature neuronal phenotypes GCmat1 and GCmat2 had completely shut off all immature markers and expressed *Ntng1*.

The cohort analysis allowed to define the time course for cluster onset (Fig. 2C and fig. S1G). RGLs were detected at early time points and declined sharply in w8, in line with a depletion of the *Ascl1*-expressing stem cell pool bearing limited proliferative capacity and self-renewal (*25*, *30*). The observation of a similar size of the cluster #1 population at distinct time points, as opposed to the gradual decrease of RGLs, suggests that the astrocytic partition is a rather pure one. NPCs were scarce, as expected, based on their fast division and differentiation and mostly present in w1 and w2. Neuroblasts were distinguished by their early onset and rapid disappearance. Immature and young aGCs appeared sequentially with distinctive temporal progression. GCimm1 primarily emerged in w1 and reached a maximum in w2, while GCimm2 was observed in w2 and w4. GCyoung was uniquely found in w4, which strongly suggests that this cluster corresponds to developing aGCs undergoing the critical period of enhanced plasticity. GCmat1 was primarily observed in w8. Although dataset 1 was arranged in a continuous pathway with important changes in cluster composition from w2 to w4 and w8, additional transitions occurring at intermediate times might have been oversighted. To improve time resolution, we collected dataset 2 with cohorts at 2, 3, 4, 5, and 8 weeks (w2 through w8; fig. S2, A to C). We used Seurat functionality to transfer cluster labels from dataset 1 to nuclei belonging to dataset 2 (fig. S3, A and B; see Materials and Methods). To assess the biological reproducibility between both datasets, the R package Batchelor was applied, and the integration between datasets was analyzed in a batch-corrected expression space (Fig. 2E). Clusters in dataset 2 maintained overall properties including DEGs through transitions, timing of appearance of the distinct clusters, and expression of canonical markers, although with a somewhat slower pace (figs. S2, D and E, and S3C). No nuclei were assigned as NB2, in line with the prominent w1 cohort composition of this cluster in dataset 1 (fig. S1G). The onset of GCimm2 was delayed compared to GCimm1, similarly to dataset 1. GCyoung was again dominant in w4, and GCmat1 was maximally expressed at w8, but it was also present at w5, suggesting an earlier appearance. Both datasets shared a high number of DEGs along transitions, supporting their reproducibility at the transcriptional level (fig. S2F).

This transcriptomic analysis describes the continuous trajectory of a unique neuronal type, the aGC, through distinct stages from RGL to GCmat. These multiple successive clusters denote higher complexity than the predicted from functional studies and establish the molecular basis for the morphological and physiological changes occurring during aGC differentiation.

### Distinct cellular states in the trajectory from neural stem cell to mature neuron

**Fig. 3.**
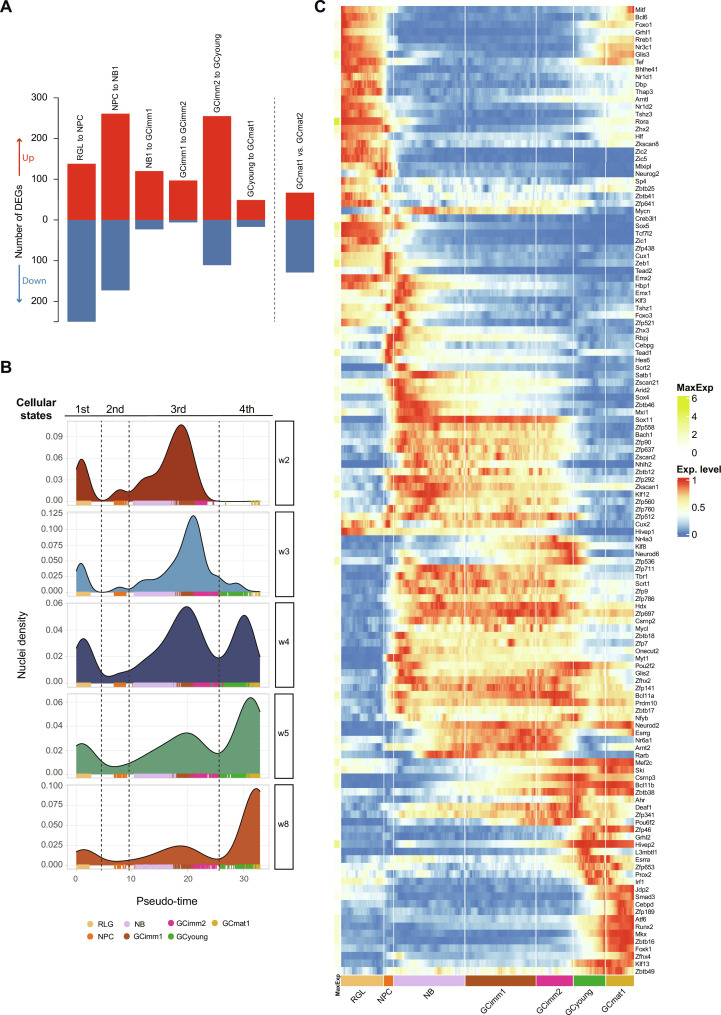
A developmental trajectory defined by four cellular states. (**A**) Differentially expressed genes (DEGs; table S2) between adjacent cluster transitions indicated above. An additional comparison (nonadjacent) is shown on the right. Up- and down-regulated genes are shown in red and blue. (**B**) Density distribution of nuclei along the pseudo-time progression for each cohort. Pseudo-time values were assigned on the basis of their profile and organized in density plots. The data revealed four cellular states with distinct cluster composition: first, RGL; second, NPC; third, NB1-GCimm1-Gcimm2; and fourth, GCyoung-GCmat1. Dashed lines depict major transitions in gene profile expression. Color codes below denote cluster identity. (**C**) Heatmap displaying the row-wise normalized expression of TFs with sharp transitions switching on/off across clusters. Genes are ordered by the pseudo-time of their switching event. Maximum expression (MaxExp; colored scale on the right) is shown on the left. Pseudo-color scale on the right denotes mean expression level. Color-coded clusters are indicated below. All data in the figure correspond to dataset 2.

The sequential pathway from RGL to NPC and then NB1 involved profound cell biological transitions, from a quiescent to a proliferating state and then to a postmitotic neuroblast. In both datasets 1 and 2, these transitions displayed the highest numbers of DEGs, particularly in terms of transcript shutdown. Unexpectedly, a high number of DEGs with marked gene down-regulation were also observed in the passage from GCimm2 to GCyoung (Fig. 3A and fig. S4A).

To better understand the dynamics of the developmental pathway, we assigned pseudo-time values to all nuclei along the continuous trajectory from RGL to GCmat1 (see Materials and Methods) and organized the datasets to obtain one density plot for each cohort (Fig. 3B and fig. S4B). Pseudo-time density plots displayed four peaks separated by clear valleys: one peak containing RGLs, another peak containing NPCs, a longer window encompassing all immature neurons (NB1 through GCimm2), and a last peak including GCyoung and GCmat1. The valleys were precisely coincident with the largest transitions resulting from the DEG analysis. The topography of these density plots seems to define four putative states along the pathway of neuronal differentiation.

To unveil the biological significance underlying these states, we analyzed the profile of TF expression. Aligning TFs in accordance to their expression onset or shutoff also revealed four distinct segments that were readily observed in the resulting heatmaps and corresponded to the peaks described above (Fig. 3C and fig. S4C). TF expression displayed different patterns, with a majority restricted to the RGL cluster (such as *Etv4*, *Etv5*, *Hes5*, and *Gli1*), others remaining in NPCs (*Ascl1*, *Sox6*, *Sox9*, and *Fezf2*), and multiple TFs covering the trajectory from NB to GCimm2 (*Sox4*, *Sox11*, *Tbr1*, and *Klf12*). Other TFs remained silent through development and became expressed in GCyoung (*Atf6*, *Runx2*, *Mkx*, and *Foxk1*). A subset of TFs expressed in RGLs shuts down in NPCs and was up-regulated again in GCyoung and GCmat1 (*Foxo1*, *Glis3*, *Bcl6*, and *Hlf*). Within each segment, cell clusters displayed little modifications in TFs.

The switches in TF expression, the critical transitions delimited by DEGs, and the valleys separating the peaks in the pseudo-time plots were all coincident within the same three transitions, revealing four crucial states that establish the developmental trajectory. Four distinct cellular states are thus defined: one corresponding to RGLs, another containing NPCs, a third state enclosing immature neurons from NB1 to GCimm2, and a fourth one encompassing GCyoung and GCmat1.

### Building neuronal function through effector molecules

**Fig. 4.**
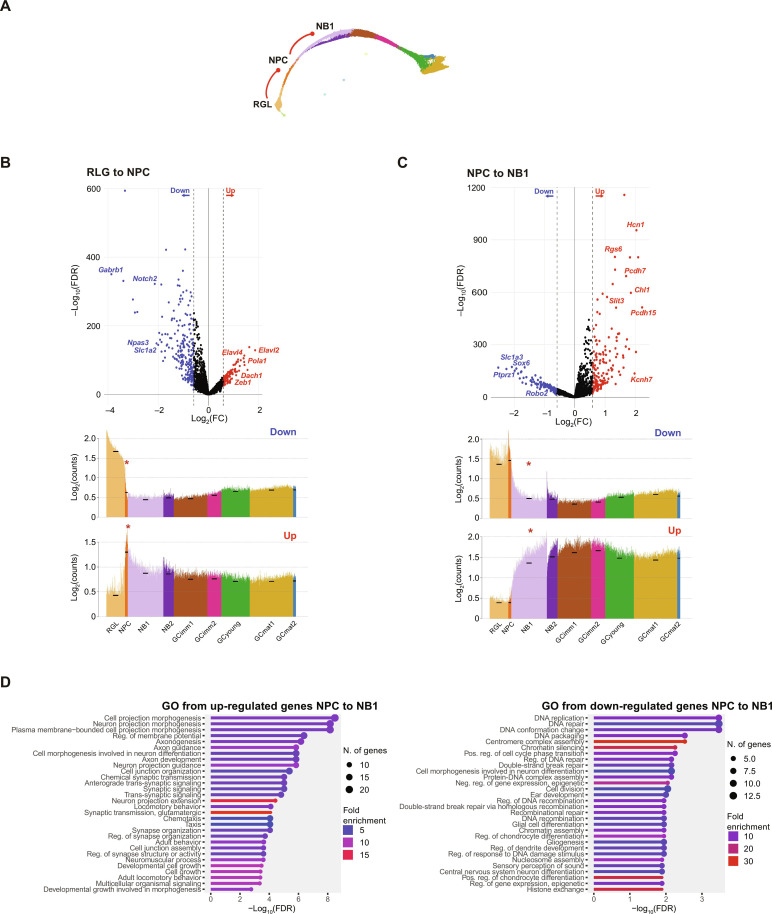
Major switches in gene profiles and biological processes during early neuronal commitment. (**A**) Schematic mKNN graph depicting the analyzed transitions (red lines). (**B** and **C**) Analysis of RGL to NPC (B) and NPC to NB1 transitions (C). Top panels: Volcano plots showing differential expression analysis [fold change (FC) ≥ 1.5 or ≤ −1.5 and false discovery rate (FDR) ≤ 0.05] displaying relevant gene examples. Bottom panels: Spike plots displaying mean expression of down- and up-regulated DEGs for all nuclei ordered according to their estimated pseudo-time value. Black dashes show the mean expression for each cluster, and the red asterisks highlight the analyzed transitions. (**D**) Top Gene Ontology (GO) biological processes for the enrichment analysis of DEGs in the transition from NPC to NB1. FDR cutoff = 0.05. All data in the figure correspond to dataset 1. DEGs are listed in table S3.

The transition from the first (RGL) to the second (NPC) cellular state switched off about 200 genes that remained silenced thereafter, reflecting a transformation from a multipotent program toward active proliferation (Fig. 4, A and B). NPC was the only mitotic state, expressing multiple cell-cycle genes and displaying an early neuronal commitment revealed by the expression of *Eomes*, *Sox4*, *Sox11*, and *Igfbpl1* (Fig. 2D and fig. S5A). The end of cell division delimits the exit from the second state toward postmitotic differentiation, starting with two types of neuroblasts, NB1 and NB2, expressing maximal levels of *Dcx* and *Igfbpl1* (Fig. 4C). This transition to the third state up-regulated transcripts controlling neuronal differentiation and morphogenesis, cell-cell interaction, development and guidance of neuronal projections, and synaptic organization (Fig. 4D).

**Fig. 5.**
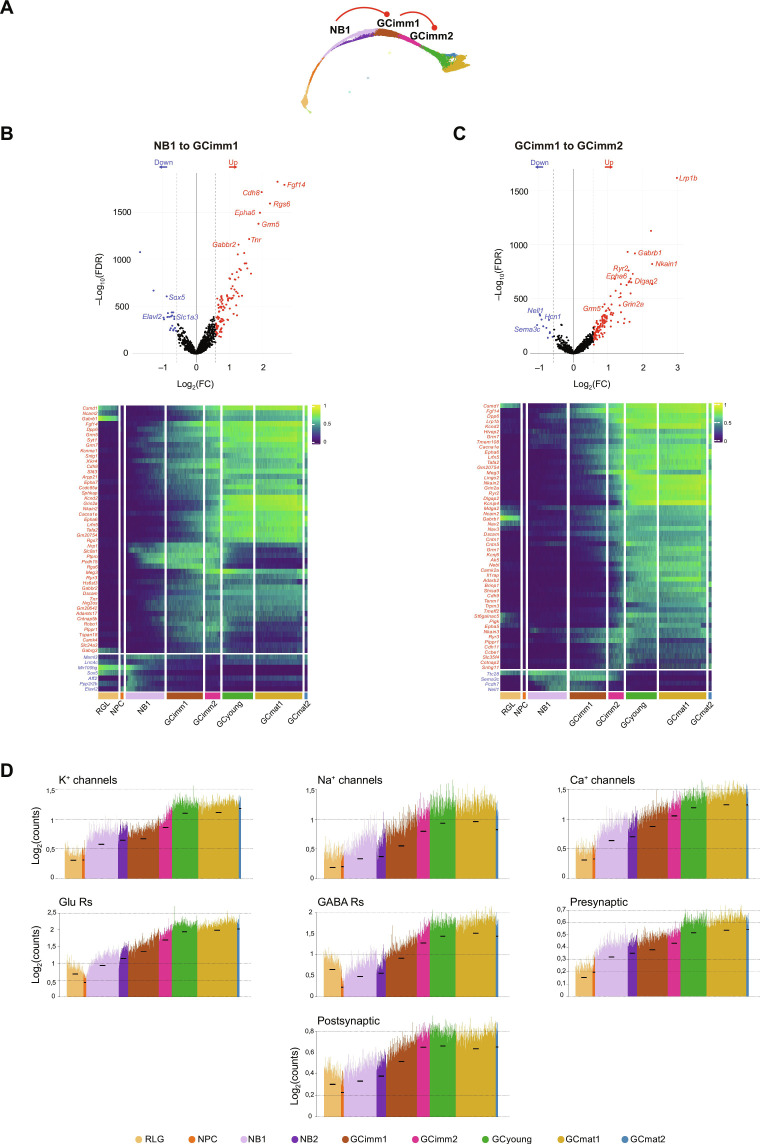
Up-regulation of effector genes required for neuronal function in immature aGCs. (**A**) Schematic mKNN graph depicting the analyzed transitions (red lines). (**B**) Analysis of NB1 to GCimm1, and (**C**) GCimm1 to GCimm2 transitions. Top panels: volcano plots showing differential expression analysis (FC ≥ 1.5 or ≤ −1.5 and FDR ≤ 0.05) with relevant gene examples. Bottom panels: Heatmap showing the row-wise normalized expression of up- (red) and down-regulated DEGs (blue) across clusters (top 50 genes per transition). Pseudo-color scale on the right denotes mean expression level. DEGs are listed in table S3. (**D**) Spike plots showing the mean expression of genes relevant for neurotransmission (table S4). Nuclei were ordered according to their estimated pseudo-time value. Black dashes show the mean expression for each cluster. All data in the figure correspond to dataset 1. Rs, receptors.

The third state comprises several immature neuronal clusters, from NB1 to GCimm2, that share the expression of canonical markers of early development: *Dcx*, *Sox4*, *Sox11*, *Igfbpl1*, and *Rgs6* (Fig. 2D and fig. S3C). Transitions among clusters in this state primarily involved up-regulated gene expression with few down-regulated transcripts, suggesting that aGCs continued to incorporate molecules that expanded their structural and functional capabilities (Fig. 3A and fig. S4A). In general, effector genes that showed an onset of expression in NB1 increased steadily and were maintained until maturation (Fig. 4C). This was the case for genes related to synaptic transmission such as *Cacna1e*, *Camk4*, *Cdh8*, *Fgf14*, *Gabrb1*, *Gabrg3*, *Grin2a*, *Grm5*, *Grm7*, *Kcnma1*, and *Syt1* (Fig. 5, A to D).

NB2 constituted a parallel pathway diverging from NB1 toward GCimm1. In the progression from w1 to w2, the NB2 population dropped twice as fast as NB1, suggesting a shorter half-life (Fig. 2C and fig. S1G). The NB2 population expressed the canonical markers for the neuroblast stage *Calb2*, *Tac2*, and *Rgs6* (Fig. 2D). Compared to NB1, NB2 displayed about 50 additional transcripts with almost no reduction in gene expression (figs. S4A and S5B). Several transcripts continued to increase in GCimm1 and GCimm2, suggesting that NB2 might represent a more advanced developmental stage. Some of these up-regulated genes are related to the assembly of glutamate receptors (*Grin2a*, *Grid1*, and *Grip1*), axon guidance (*Robo2*, *Sema3c*, and *Unc5d*), and cell-cell interaction (*Ephb1*, *Cdh4*, *Pcdh7*, and *Nrxn3*) (Fig. 5D and fig. S5C).

The transition from NB1 to GCimm1 displayed a marked increase in GABA and glutamate receptor subunits (*Gabrb1*, *Grm5*, *Grm7*, *Gabbr2*, *Gabrg3*, and *Grin2a*), voltage-gated ion channels (*Kcnma1*, *Kcnd2*, and *Cacna1e*), and axon guidance (*Robo1*, *Nrp1*, *Epha7*, *Dscam*, *Ncam2*, *Epha6*, *Slit3*, and *Ptpro*), critical for the establishment of membrane excitability, neuronal growth, and synaptic transmission (Fig. 5, B and D).

GCimm1 and GCimm2 shared specific markers that were previously described for immature GCs (fig. S6, A and B) (*28*). Besides these general similarities, GCimm2 displayed a distinct profile indicating a more advanced developmental stage, including genes related to synapse formation and plasticity, such as *Camk2a* and multiple glutamate receptor subunits: *Grm1*, *Grid1*, *Gria3*, *Grin2a*, *Grm5*, and *Grm7* (Fig. 5, C and D) (*36*–*38*). These findings suggest that GCimm2 initiates a program for glutamatergic synaptogenesis, which, in electrophysiological studies, was shown to begin in 2-week-old aGCs.

The early clusters described above—RGL, NPC, and neuroblasts—reflect different cellular states that bring neural stem cells toward postmitotic neuronal differentiation. In the transitions occurring within the third state (NB to GCimm1 and then to GCimm2), developing aGCs incorporate molecules that expand their functional capabilities, with little transcript shutdown.

### Molecular blueprint of neurotransmission and activity

**Fig. 6.**
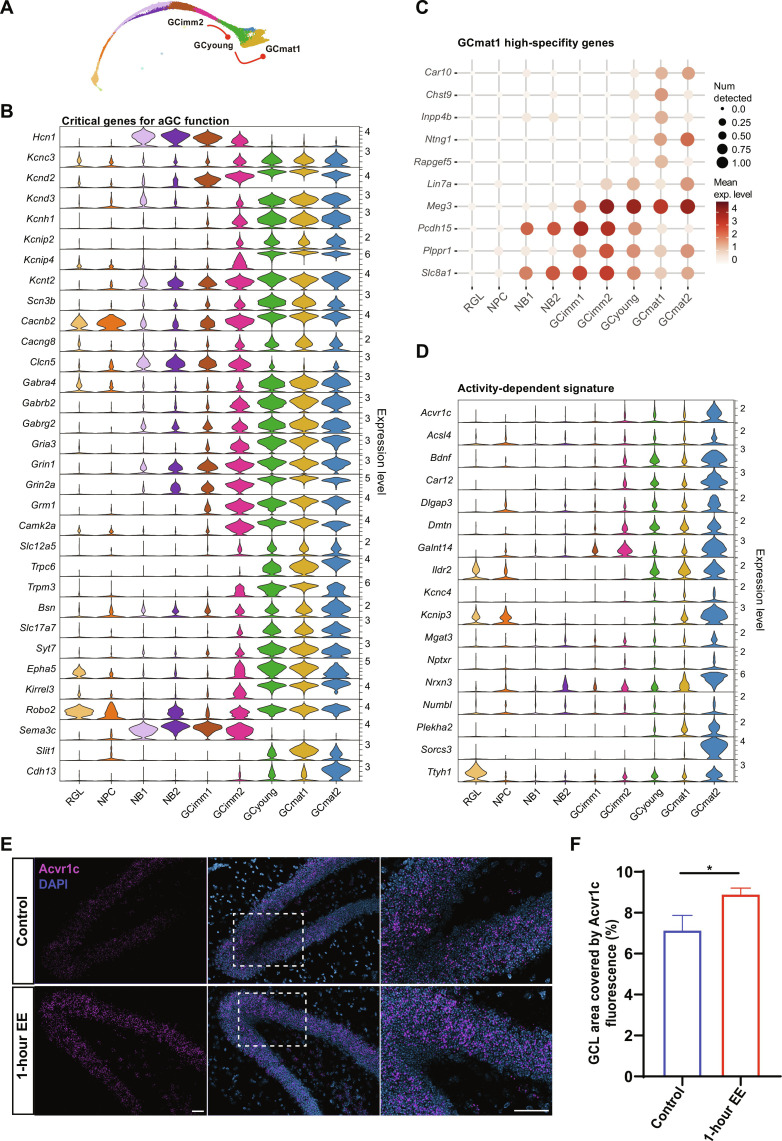
Molecular blueprint of mature aGCs. (**A**) Schematic mKNN graph depicting the analyzed transitions (red lines). (**B**) Violin plots showing example genes critical for aGC function. (**C**) Dot plot exhibiting genes selectively up- and down-regulated in GCmat1. Scales on the right correspond to mean expression levels (color) and to the fraction of nuclei expressing at least one transcript count in the cluster (dot size). (**D**) Violin plots depicting the expression of transcripts that are known to be induced by activity (*40*). All data in the figure correspond to dataset 1. (**E**) In situ hybridization reveals the expression of the activity-induced gene *Acvr1c* in the granule cell layer (GCL). Mice were exposed to an enriched environment for 1 hour (EE) or remained in a home cage (control), and hippocampi were frozen 5 hours later. Representative images depict *Acvr1c* (pink) expression [4′,6-diamidino-2-phenylindole (DAPI), blue]. Scale bars, 50 μm. Dashed box areas shown on the right panels depict areas of the GCL expressing Acvr1c, which exclude the subgranular zone. (**F**) GCL area occupied by Acvr1c fluorescence in control and EE mice. *n* = 10 sections per three mice (control) and *n* = 9 sections per three mice (EE). Asterisk (*) denotes *P* < 0.05 after Mann-Whitney test. Data depicts means ± SEM.

The passage from GCimm2 to GCyoung revealed a major switch that marked the end of the immature period and the beginning of the fourth state (Figs. 3 and 6, A and B, and fig. S4). This transition exhibited a large number of DEGs, highlighting the complexity required to reach neuronal maturation. Multiple transcripts that had reached maximal expression during early development shut down in GCyoung: *Adamts18*, *Dcx*, *Hcn1*, *Igbpl1*, *Robo1*, *Rgs6*, *Sema3c*, *Sema3e*, *Sox4*, and *Sox11*. To better understand the functional changes involved in this transition, we performed enrichment analysis of Gene Ontology (GO) terms (fig. S7). Genes related to axonal growth and development shut down, while those orchestrating postsynaptic organization, transmission, and plasticity appeared during this period. Kirrel3, a synaptic adhesion molecule required for the formation of mossy fiber contacts onto GABAergic interneurons, providing feed-forward inhibition onto CA3 pyramidal cells, is up-regulated during this transition (Fig. 6B) (*39*). Furthermore, GCyoung showed plateau levels in typical effector genes related to excitability and neurotransmission (Fig. 5D). These transcripts included the vesicular glutamate transporter VGLUT1 (*Slc17a7*), the K^+^/Cl^−^ cotransporter KCC2 responsible for the GABA switch (*Slc12a5*), and *Camk2a* and *Grin2a*, crucial for synaptic plasticity (Fig. 6B). These features are entirely consistent with prior electrophysiological characterizations of hyperexcitable 4-week-old aGCs (*14*–*16*, *18*, *20*).

The GCyoung and GCmat1 clusters, corresponding to the fourth state, were observed at the w4 and w8 time points, respectively, which is coincident with the interval at which aGCs become functionally mature (*3*). This transition was characterized by a few DEGs with subtle changes in their expression level (fig. S8, A and B). A small group of the DEGs constituted markers of GCmat1: *Car10*, *Chst9*, *Inpp4b*, *Ntng1*, and *Rapgef5* (Fig. 6C). Other genes were specifically down-regulated in GCmat1: *Lin7a*, *Meg3*, *Pcdh15*, *Plppr1*, and *Slc8a1*. Although 4- and 8-week-old aGCs are known to be functionally distinct, GCyoung and GCmat1 clusters displayed similar expression profiles. This observation indicates that physiological maturation is influenced by additional factors other than their transcriptomic signature.

GCmat2 was a small but sharply defined cluster that contained cells belonging to cohorts w4 onward in both datasets and exhibited a high number of DEGs when compared to GCmat1 (Fig. 3A and fig. S4A). GCmat2 displayed a set of well-defined markers whose expression has been linked to activity of dentate GCs: *Acvr1c*, *Sorcs3*, *Nrxn3*, and *Kcnip3*, among others (Fig. 6D) (*40*). *Bdnf*, a neurotrophin whose synthesis and secretion depend on neuronal activity, was only expressed in this cluster (*41*, *42*). Moreover, *Rgs6* (a marker for immature neurons) has been reported to increase in aGCs after running, and it is up-regulated in GCmat2 (*43*). *Cdh13*, a negative regulator of inhibitory synaptogenesis in the hippocampus, was also found to be expressed in GCmat2 (Fig. 6B) (*44*, *45*). To confirm this activity-dependent signature, mice were exposed to an enriched environment for 1 hour, a condition that enhances neuronal activity in the GC layer (*46*). This stimulus resulted in a marked increase in the expression of the *Acvr1c* receptor monitored by immunofluorescent in situ hybridization (Fig. 6, E and F). Therefore, GCmat2 was classified as a cluster containing neurons belonging to the w4 to w8 time points that were likely to be electrically active before isolation.

**Fig. 7.**
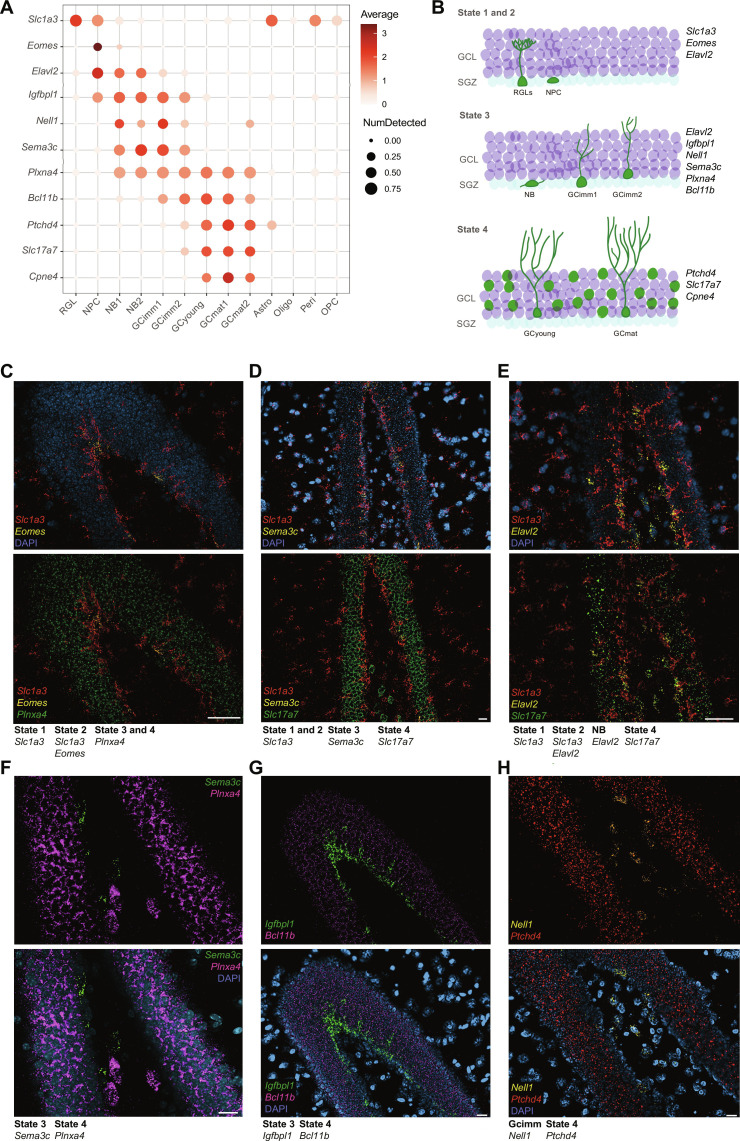
Spatial validation of cellular states and cluster markers by RNA in situ hybridization. (**A**) Dot plot exhibiting markers along clusters. Scales on the right correspond to log_2_ (mean gene expression) (pseudo-color) and to the fraction of nuclei expressing at least one transcript count in the cluster (dot size). All data corresponds to dataset 1. (**B**) Schematic representation of cells belonging to different clusters (green) distributed within the GCL (purple) and subgranular zone (SGZ; pale blue). Marker genes for each cell state are listed on the right. (**C** to **H**) In situ hybridization of dentate gyrus sections from 6- or 7-week-old mice. Example of deconvolved images. A distinctive color was assigned to each marker and the specific gene combinations defining cellular states are listed beneath the panels. State 1, 2, or 3 markers are expressed in cells located near the SGZ. State 4 markers are expressed by all mature GCs, not limited to aGCs. Therefore, RNA molecules are distributed throughout the GCL. DAPI (blue) was used to label cell nuclei. Scale bars, 50 μm

Last, some of the markers were validated by fluorescence in situ hybridization (Fig. 7). *Slc1a3*, *Sema3c*, *Nell1*, *Igfbpl1*, *Elavl2*, and *Eomes* are markers for state 1, 2, or 3, and their expression was confined to the inner GC layer, where early aGC stages are typically found (*47*–*49*). *Cpne4*, *Slc17a7*, *Ptchd4*, *Bcl11b*, and *Plxna4* are markers for the fourth state and were expressed by most mature GCs in the entire GC layer, including aGCs (Fig. 7, A and B). Marker specificity was further tested by their combined expression, showing restricted labeling patterns of cell states or clusters with distinct spatial localization (Fig. 7, C to H).

### A transcriptional switch underlying neuronal maturation

To identify master transcriptional regulators for the developmental trajectory, we evaluated the expression of TFs and their target genes (regulons), corresponding to the transitions between cellular states. This analysis was performed using *SCENIC* (see Materials and Methods). In particular, we considered regulon specificity scores (RSSs) to analyze changes in regulon activity between consecutive states (*50*). The transition from peaks 1 to 2 and from peaks 2 to 3 revealed genes involved in biological programs that enabled switching from quiescent (*Sox6*, *Pax6*, *Sox5*, and *Sox9*) to proliferative (*Eomes*, *E2f1*, *E2f3*, *E2f6*, and *E2f7*) and then postmitotic states (*Neurod2*, *Sox4*, and *Sox11*) (fig. S9A).

**Fig. 8.**
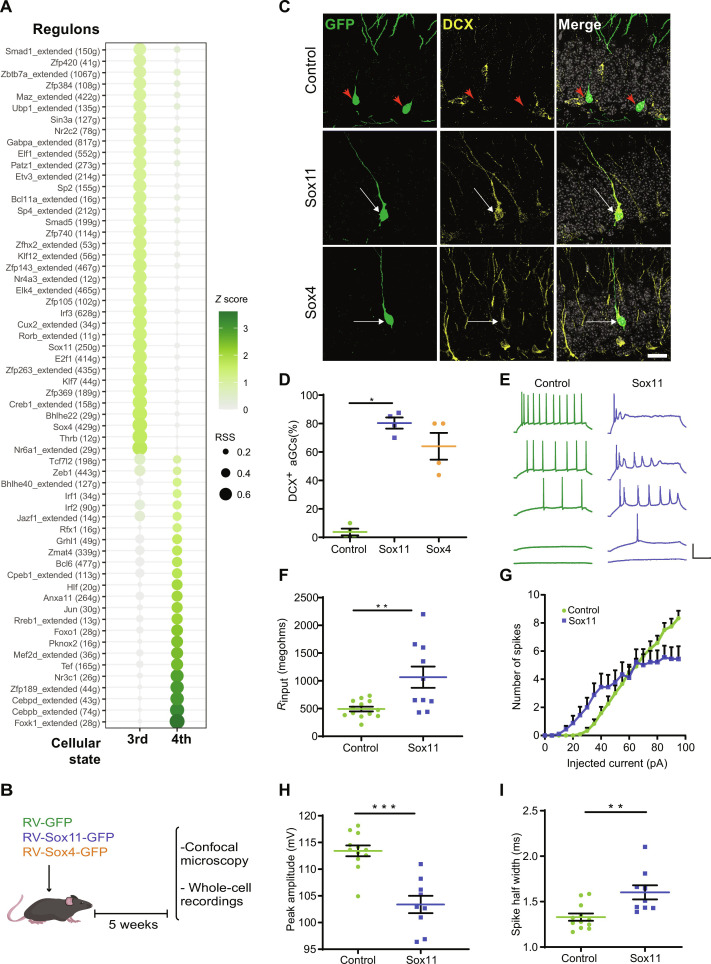
Overexpression of SoxC TFs precludes aGC maturation. (**A**) Regulon activity analysis based on the expression levels of TF targets comparing the third and fourth states (dataset 2). Each regulon contains targets genes identified by SCENIC according to the conserved DNA binding motifs in regulatory regions. Regulon sizes are shown in parentheses. Extended regulons include targets inferred by binding motif similarity. Regulon compositions are listed in table S5. RSS, regulon specificity score (*50*). The *z*-score color scale depicts standardized expression activity values. (**B**) New aGCs were labeled in adult mice by stereotaxic delivery of RV-GFP, RV-Sox11-GFP, or RV-Sox4-GFP, as indicated. aGCs expressing GFP were studied 5 weeks after injection. (**C**) Representative single confocal planes displaying aGCs expressing Sox11-GFP, Sox4-GFP, or control GFP (green, arrowheads) and DCX (yellow). Colocalization is highlighted by white arrows. Scale bars, 20 μm. (**D**) Proportion of GFP^+^ neurons expressing DCX, normalized to the total number of GFP^+^ neurons. *n* = 80 aGCs from four mice for each condition. *P < 0.02, Kruskal-Wallis test. (**E** to **I**) Whole-cell recordings in acute slices from aGCs expressing RV-GFP or RV-Sox11-GFP. (E) Representative voltage traces elicited by current steps of increasing amplitude: 5, 20, 40, 60, and 95 pA. Scale: 50 mV, 100 ms. Resting potential (*V*_resting_ ) kept at −80 mV. (F) Input resistance (*R*_input_). GFP-aGCs, *n* = 13; Sox11-aGCs, *n* = 10 (4 mice/condition). **P < 0.005, *t* test. [(G) to (I)] Action potentials elicited by injected current steps (500 ms). Plots show the total number of spikes per pulse (*P* < 0.001) (G), the amplitude of the first spike (H), and the spike half width, measured at half-maximal amplitude (I). Control GFP-aGCs, *n* = 11, and Sox11-aGCs, *n* = 9 (four mice per condition). **P < 0.005 and ***P < 0.001. Data are shown as means ± SEM.

The comparison between the third and the fourth states (immature to mature aGCs) revealed >20 regulons displaying selective expression in the third state, with high levels for the *SoxC* family genes *Sox4* and *Sox11* (Fig. 8A and fig. S9B). Regulons present in the fourth state included *Bcl6*, *Foxo1*, *Anxa11*, *Tef*, and *Hlf* (fig. S9C). All of these TFs have been shown to control signaling pathways regulating neuronal homeostasis (*51*–*54*). *Sox4* and *Sox11* have been shown to play a critical role in early differentiation and neuronal growth, both during perinatal development and in adult neurogenesis (*55*, *56*). However, their participation during late neuronal maturation remains unknown. The shutdown of these *SoxC* TFs in the last neuronal state suggested two possibilities: (i) they are not needed for the final transition and their down-regulation results as a secondary effect of a global transcriptional program and (ii) their absence might be required for developing neurons to transition to the mature state.

To discriminate between these possibilities, we investigated the effect of the sustained expression of *Sox4* or *Sox11* on aGC differentiation. RV-Sox4-GFP or RV-Sox11-GFP was delivered to the dentate gyrus of adult mice to infect mitotic NPCs, coincident with the onset of endogenous expression of *Sox4* and *Sox11* (Fig. 8B). The impact of TF overexpression in the neuronal progeny was studied after 5 weeks, at which time new aGCs become mature. As revealed by immunofluorescence imaging, the immature neuronal marker DCX was absent in control aGCs, but it was observed in 60 to 80% of neurons overexpressing Sox4 or Sox11, suggesting that cells failed to down-regulate DCX upon Sox4/11 overexpression (Fig. 8, C and D). In addition, mature aGCs overexpressing Sox11 [the SoxC member with highest transactivation potential; (*57*)] revealed immature properties such as high membrane resistance and excitability, as well as impaired capacity to fire repetitive action potentials in acute hippocampal slices (Fig. 8, E to I). Together, these results indicate that aGCs with sustained expression of Sox4 or Sox11 maintain immature features (*58*, *59*). We therefore conclude that shutdown of *SoxC* TFs is a necessary condition for developing aGCs to become mature. The transition from the immature to the mature state constitutes a critical transformation driven by a strong and unique transcriptional switch that shuts down molecular cascades promoting cell growth and activates a final state that seems to be dominated by neuronal homeostasis.

## DISCUSSION

The molecular basis underlying the differentiation and functional integration of aGCs has remained largely unknown. Previous work has captured aspects of adult neurogenesis by means of transcriptomic profiling with a focus on the early molecular cascades that distinguish quiescent from active RGLs and NPCs (*26*, *60*). Subsequent research has conveyed the notion of a linear trajectory reaching the early neuroblast stages (*27*). More recently, a study reported that immature GCs obtained from the perinatal, postnatal, and adult hippocampus display similar transcriptional profiles (*28*). In this work, we performed a comprehensive study encompassing differentiation and functional maturation of aGCs. The strategy involved the analysis of cohorts of aGCs at six time points that allowed us to assign precise time stamps to the developmental trajectory. In general, the dynamics of gene expression profiles matched very closely with previous descriptions of developing aGCs obtained using immunohistochemical, morphological, and electrophysiological approaches. These datasets now provide a thorough description of distinctive molecular states underlying neuronal differentiation in the adult hippocampus, assigning specific profiles to the intermediate stages, revealing TFs controlling the process, and the effector genes that convert those programs into neuronal function.

The different phases of adult neurogenesis were previously characterized by the expression of stage-specific markers defined by immunolabeling. RGLs gave rise to NPCs and, subsequently, to neuroblasts that were thought to keep limited proliferative capacity (*61*, *62*). Instead, our results reveal that NPCs are the only mitotic cluster in aGC differentiation, and neuroblasts are committed postmitotic cells (*28*). Two types of neuroblasts were observed: NB1 arising from NPCs, and NB2 shedding from NB1, forming a parallel pathway reaching GCimm1. GCimm1 and GCimm2 were composed mostly of 2- to 4-week-old aGCs and expressed immature neuronal markers. The transition to GCimm2 up-regulated >100 genes, including those related to glutamatergic transmission, which is known to begin by the second developmental week (*47*, *63*–*65*). This finding indicates that GCimm2 may be the molecular transition that accompanies the onset of glutamatergic synaptogenesis.

Approaching the GCyoung stage involved substantial profile changes (250 to 350 DEGs in both datasets) that were only comparable in magnitude to the transitions from RGL to NPC and from NPC to NB1. Multiple effector genes reached plateau levels at GCyoung, including those required for neurotransmission and plasticity, from the presynaptic vesicular glutamate transporter *Slc17a7* and vesicular release *Syt7* to postsynaptic glutamate and GABA receptors. Because GCyoung contained predominantly w4 cells, we propose that aGCs in this cluster correspond to 4-week-old aGCs with enhanced excitability and synaptic plasticity, described extensively in the literature (*14*–*16*, *18*, *20*, *64*). Despite containing the fundamental building blocks required for neuronal function, 4-week-old GCs are not yet mature. Mature aGCs (>8 weeks) exhibit maximal glutamatergic synaptic strength, mature GABAergic inhibition, and reduced excitability (*18*, *58*, *66*, *67*). Few transcripts were found to be selectively changed in the transition from GCyoung to GCmat1, suggesting that the GCyoung cluster is the early phase in a final step of maturation that becomes consolidated over time.

**Fig. 9.**
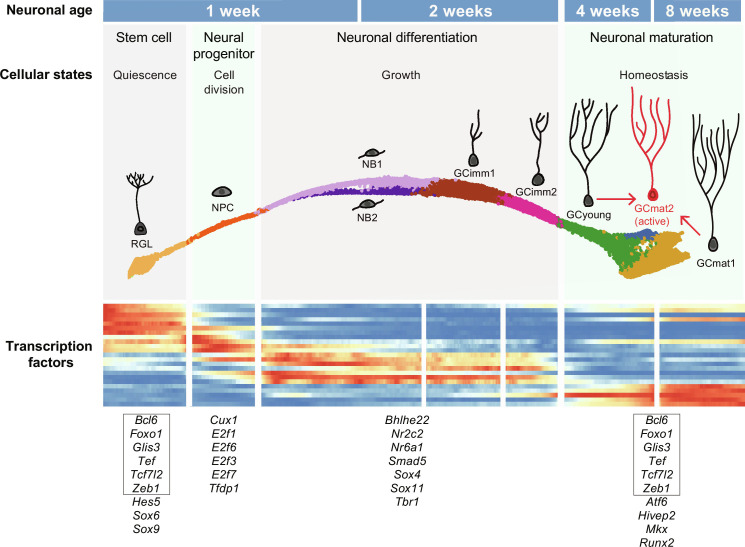
Schematic representation of cell states during aGC maturation. Transcriptomic profiling of developing aGCs unveils a molecular continuum divided in nine clusters grouped into four cellular states, precisely defined by the activation and deactivation of specific transcription factors (TFs). During the developmental period lasting about 8 weeks, distinct TFs and effector genes orchestrate the biological process characteristics of these states. Stem cells abandon the quiescence state to become mitotic (cell division state). Dividing NPCs rapidly acquire a neuronal fate, entering a state of cellular growth. These passages occur during the first developmental week. Postmitotic neurons express a large set of TFs driving differentiation. By the fourth week, growth achieves a plateau and previously expressed programs shut down to activate a set of TFs driving homeostatic control of neuronal function (homesotasis state). During this final interval, aGCs are known to become fully integrated in the preexisting network. The heatmap (bottom panel) depicts the expression of the example TFs listed below (pseudo-color scale is the same as in Fig. 3C). Squared TFs were expressed both in the quiescent and homeostatic states.

Analysis of DEGs, pseudo-time trajectory, and TF expression revealed three critical transitions defining four cellular states: RGL, NPC, immature neurons (NB1 to GCimm2), and a mature state (GCyoung and GCmat; Fig. 9). Regulon analysis revealed that *Sox4* and *Sox11* regulons played critical roles as organizers of the immature state. These TFs were known to be involved in the early steps of differentiation during perinatal and adult neurogenesis (*55*, *56*). We now demonstrate that shutdown of *SoxC* TFs is a critical mechanism required for achieving terminal differentiation.

TFs and regulons dominating the fourth state included *Bcl6*, *Foxo1*, *Klf9*, *Hlf*, *Tef*, *Anxa11*, and *Atf6*. The known functions of these TFs suggest that they might coordinate a fine homeostatic regulation. For instance, deletion of *Foxo* family TFs has been shown to impair autophagy, leading to aberrant dendritic growth and increased spine density in aGCs (*51*). *Bcl6* acts, in part, through Foxo1 to control neuron survival (*54*). *Klf9* knockout resulted in increased spine density and impaired functional maturation in aGCs (*68*). Moreover, genetic ablation of *Hlf* and *Tef* in cultured neurons triggered a disproportionate up-regulation of input excitation when deprived of activity (*52*). This evidence points to a central role of these TFs in the homeostatic regulation of excitatory neuronal connectivity. *Anxa11* and *Atf6* have been associated with calcium homeostasis (*53*, *69*, *70*). *Foxo1*, *Tef*, *Atf6*, and *Mkx* share a common downstream TF, *Hivep2* (Schnurri-2), whose knockout provoked global alterations in the morphology of dentate gyrus GCs (*71*). Last, *Glis3*, a TF that was selectively expressed in RGLs and then up-regulated in GCmat1, has been associated with autophagy and with the regulation of neuronal growth and complexity (*72*). Together, the regulatory roles of these TFs and their late expression support a hypothesis whereby neuronal maturation is orchestrated by shutting down molecular cascades promoting differentiation and by awaking signaling pathways controlling cellular homeostasis. The data presented here opens a new scenario to unravel the temporal dynamics involving master transcriptional regulators that control neuronal maturation in the healthy adult brain and will serve as the basis to identify mechanisms contributing to aberrant circuit remodeling in brain disorders.

## MATERIALS AND METHODS

### Animals

Male and female C57Bl/6J wild-type and genetically modified mice (6 to 8 weeks of age) were housed at four to five mice per cage in standard conditions. For adult neurogenesis snRNA-seq experiments, *Ascl1^CreERT2^* [*Ascl1^tm1(Cre/ERT2)Jejo/J)^*] mice were crossed to *CAG^floxStop-Sun1/sfGFP^* [B6.129*-Gt(ROSA)26Sort^m5.1(CAG-Sun1/sfGFP)Nat/MmbeJ^*] conditional reporter line to generate Ascl1^CreERT2^;CAG^floxStop-Sun1/sfGFP^ mice, which were used to reliably target adult-born GC nuclei (*30*, *31*, *73*). TAM induction (120 μg/g, four injections in two consecutive days) resulted in the expression of Sun1-sfGFP in the nuclear envelope of Ascl1^+^ cell progeny. Mice were anesthetized (150 μg of ketamine/15 μg of xylazine in 10 μl of saline per gram) and euthanized at the indicated times after TAM induction: 1, 2, 4, and 8 weeks for dataset 1 and 2, 3, 4, 5, and 8 weeks for dataset 2. Mice were maintained in C57BL/6J background. Experimental protocols were approved by the Institutional Animal Care and Use Committee of the Leloir Institute (CICUAL-FIL 85), according to the Principles for Biomedical Research involving animals of the Council for International Organizations for Medical Sciences and provisions stated in the *Guide for the Care and Use of Laboratory Animals*. Leloir Institute is approved as a foreign facility by the Office of Laboratory Animal Welfare of the US National Institutes of Health (F18-00411).

### Tissue dissection

Mice were deeply anesthetized (ketamine/xylazine, as described above), and brains were carefully removed and placed into ice cold Earl’s balanced salt solution (117 mM NaCl, 5.4 mM KCl, 1 mM NaH_2_PO_4_, 26 mM NaHCO_3_, 5.6 mM glucose, 1.8 mM CaCl_2_·2H_2_O, and 0.8 mM MgSO_4_) with trehalose (5% v/v) and kynurenic acid (0.8 mM_ (*74*). Dissection solution was equilibrated in 95% O_2_/5% CO_2_ before use. The brain was cut along the longitudinal fissure, and the regions posterior to lambda were cut off. Under a dissection microscope and upon removal of the diencephalon, the medial side of the hippocampus was exposed. The dentate gyrus was isolated by inserting a sharp-needle tip and sliding it superficially along the septotemporal axis of the hippocampus. The dissected tissue was placed in a 0.5-ml microcentrifuge tube with a minimal amount of medium, flash-frozen on dry ice, and stored at −80°C until use.

### Nuclei isolation and FACS sorting

Nuclei were isolated as previously described with several modifications (*75*). Dounce tissue grinder and pestles were sequentially washed with 100% EtOH, ribonuclease (RNAse) Zap (Sigma-Aldrich, catalog no. R2020), and two to three rounds with RNAse-free water and lastly rinsed with EZ Lysis Buffer (Sigma-Aldrich, catalog no. NUC-101). All material and buffers were chilled on ice. The tissue was transferred to a chilled dounce prefilled with 2 ml of ice-cold EZ Lysis Buffer and homogenized slowly with 20 to 25 strokes of pestle A, followed by 20 strokes with pestle B. Suspension was transferred to a chilled tube and incubated on ice 5 min. Then, the suspension was centrifuged at 500*g* for 5 min at 4°C, and the supernatant was removed. The pellet was resuspended in 4 ml of ice-cold EZ Lysis Buffer, and, after a second round of ice incubation and centrifugation, the isolated nuclei were resuspended in 4 ml of ice-cold Nuclei Suspension Buffer (NSB) [1× RNase-free molecular biology–grade phosphate-buffered saline, 0.1% molecular biology–grade bovine serum albumin (BSA; 100 μg/ml), and RNase inhibitor (0.2 U/μl)]. Last, nuclei were centrifuged at 500*g* for 5 min at 4°C and, after removing the supernatant, were resuspended in 1 ml of NSB-Ruby (final Ruby concentration, 1:500) and filtered twice through a 35-μm cell strainer to remove as many debris as possible. Nuclei were kept on ice until sorting (Harvard University, Bauer Core Facility, BD FACSAria II) into 96-well plates, pre-coated with BSA to reduce adherence and improve recovery, containing with ~10 to 20 μl of rich NSB [with final RNase inhibitor (2 U/μl) and 1% BSA]. The final nuclei concentration was determined using a C-chip Neubauer counting chamber and Trypan Blue (1:2 final dilution). Nuclei were immediately loaded for single-cell GEM formation (10x Genomics, single-cell RNA sequencing 3′, Chromium v3.1).

### 10x Genomics Chromium

Dataset sampling for snRNA-seq experiments was carried out within a minimal time interval, processing two time points in a single experiment each day to minimize batch effects. For each time point, four dentate gyri (left hemispheres) from male and female mice were pooled. If needed to increase nuclei number, then dentate gyri from the right hemisphere were also processed (dataset 2). For snRNA-seq, nuclei were loaded in the 10x Genomics chips aiming to recover 2000 to 10,000 nuclei. cDNA amplification and library construction were done following 10x Genomics protocols. For both dataset 1 and dataset 2, libraries were generated using Chromium v3.1, quantified in BioAnalyzer, and sequenced on an Illumina HiSeq or NovaSeq. Samples were sequenced to a depth of 40 to 70,000 reads per cell.

### RNA in situ hybridization

For validation of cluster markers, mice were anesthetized, and the brains were removed and placed into the ice-cold dissection solution (described above). Hippocampi were dissected under a microscope and immediately embedded in OCT on dry ice and stored at −80°C. Sections of 14 μm covering the anterior-posterior axis of the dentate gyrus were collected in a cryostat. Sections were placed for 1 hour at −20°C and stored at −80°C until use. Fluorescent multiplex RNA in situ hybridization was performed using the RNAscope Fluorescent Multiplex Reagent Kit (Advanced Cell Diagnostics), according to the manufacturer’s instructions. Briefly, thawed sections were fixed in 4% paraformaldehyde and dehydrated in sequential incubations with ethanol, followed by 30 min of protease IV treatment. Mice were exposed to the enriched environment for 48 hours (Fig. 6E) before tissue sample preparation as described above. Appropriate hybridization probes [Advanced Cell Diagnostics, catalog nos. 429291-C1 (Acvr1c), 807961-C2 (Nell1), 441441-C1 (Sema3c), 430781-C3 (Slc1a3), 488851-C2 (Igfbpl1), 491961-C2 (Elavl2), 429641-C1 (Eomes), 474721-C1 (Cpne4), 501101-C2 (Slc17a7), 463451-C1 (Ptchd4), 413271-C1 (Bcl11b), and 515491-C2 (Plxna4)] were incubated for 2 hours at 40°C, followed by amplification steps (according to protocol) and 4′,6-diamidino-2-phenylindole (DAPI) counterstaining, and tissue was mounted with gerbatol to prevent bleaching.

### Production of viral vectors

A replication-deficient retroviral vector based on the Moloney murine leukemia virus was used to specifically transduce aGCs as done previously (*18*, *46*). Retroviral particles were assembled using three separate plasmids containing the capside (CMV-vsvg), viral proteins (CMV-gag/pol), and the transgenes: CAG-GFP, CAG-EGFP-IRES-Sox11, and CAG-EGFP-IRES-Sox4. Plasmids were transfected onto human embryonic kidney (HEK) 293T cells using deacylated polyethylenimine. HEK 293T cells were cultured in Dulbecco’s modified Eagle’s medium with high glucose, supplemented with 10% fetal calf serum and 2 mM glutamine. Virus-containing supernatant was harvested 48 hours after transfection and concentrated by two rounds of ultracentrifugation. Virus titer was typically ~10^5^ particles per microliter.

### Stereotaxic surgery for retroviral delivery

Running wheel housing was only available 2 to 3 days before surgery to maximize the number of retrovirally transduced neurons. The wheel was removed 1 day after surgery. For the stereotaxic procedure, mice were anesthetized, and retroviral particles were infused into the dorsal region of the right dentate gyrus (1 to 1.5 μl at 0.15 μl/min) using sterile-calibrated microcapillary pipettes through stereotaxic references. Coordinates from bregma: −2 mm anteroposterior, −1.5 mm lateral, and −1.9 mm ventral. A single injection site was sufficient to label an abundant number of neural precursor cells. Brain sections were prepared 5 weeks after infection for immunofluorescence or electrophysiological recordings.

### Immunofluorescence

Immunostaining was performed in 60-μm-thick free-floating coronal sections throughout the septal fraction of the hippocampus from *Ascl1^CreERT2^;CAG^floxStop-Sun1/sfGFP^* or retrovirally injected mice. Antibodies were applied in tris-buffered saline with 3% donkey serum and 0.25% Triton X-100. Immunofluorescence was performed using the following primary antibodies: DCX (rabbit polyclonal; 1:1500; Abcam) and green fluorescent protein (GFP; chicken immunoglobulin Y fraction; 1:500; Aves Labs Inc.). The following corresponding secondary antibodies were used: donkey anti-chicken Cy2 or Cy3 and donkey anti-rabbit Cy5 1:250 (Jackson ImmunoResearch Laboratories). Incubation with DAPI (10 min) for nuclear counterstain was performed. Slices were mounted and covered with gerbatol to prevent bleaching.

### Confocal microscopy

Sections from the septal hippocampus according to the mouse brain atlas (antero-posterior, −0.94 to −2.46 mm from bregma) were included (*76*). Images were acquired using an 880 LSM Airyscan microscope (Carl Zeiss, Jena, Germany). Analysis of antibody expression was restricted to cells with fluorescence intensity levels that enabled clear identification of their soma. For Sox/GFP experiments, images were acquired (40×; numerical aperture, 1.2) from 60-μm-thick sections, and colocalization was assessed using single optical planes (airy unit = 1). For RNAscope experiments (Fig. 6), images were acquired from 14-μm-thick hippocampal slices. Dot area quantification was performed in ImageJ/Fiji applying the Otsu threshold as suggested by the manufacturer (Advanced Cell Diagnostics). All experiments were done with control versus treatment conditions blind to the operator. For experiments shown in Fig. 7, images were acquired using deconvolution software (Zeiss Zen blue 3.3).

### Electrophysiology

Five weeks after retroviral injection, mice were anesthetized and brains were removed into a chilled solution containing: 110 mM choline-Cl^−^, 2.5 mM KCl, 2.0 mM NaH_2_PO_4_, 25 mM NaHCO_3_, 0.5 mM CaCl_2_, 7 mM MgCl_2_, 20 mM dextrose, 1.3 mM Na^+^-ascorbate, 0.6 mM Na^+^-pyruvate, and 4 mM kynurenic acid. Slices (400-μm thick) were cut in a vibratome (Leica VT1200S) and transferred into a chamber containing artificial cerebrospinal fluid: 125 mM NaCl, 2.5 mM KCl, 2.3 mM NaH_2_PO_4_, 25 mM NaHCO_3_, 2 mM CaCl_2_, 1.3 mM MgCl_2_, 1.3 mM Na^+^-ascorbate, 3.1 mM Na^+^-pyruvate, and 10 mM dextrose (315 mosmol). Slices were maintained with 95% O_2_/5% CO_2_ at 30°C for >1 hour before experiments started. Whole-cell recordings were performed at 23 ± 2°C using microelectrodes (3 to 5 megohms) pulled from borosilicate glass (KG-33; King Precision Glass) and filled with 120 mM K-gluconate, 20 mM KCl, 5 mM NaCl, 4 mM MgCl_2_, 0.1 mM EGTA, 10 Hepes, 4 mM tris–adenosine 5′-triphosphate, 0.3 mM tris–guanosine 5′-triphosphate, 10 mM phosphocreatine, Alexa Fluor 488 or 594 (10 μg/ml; Invitrogen), pH 7.3, and 290 mosmol. Recordings were obtained using an Axopatch 200B amplifier (Molecular Devices), digitized (Digidata 1322A), and acquired at 10 kHz into a personal computer using the pClamp 9 software (Molecular Devices). Recorded neurons were visually identified in the GC layer by fluorescence (fluorescein isothiocyanate fluorescence optics; DMLFS, Leica) and infrared differential interference contrast videomicroscopy. Input resistance was obtained from current traces evoked by a hyperpolarizing step of 10 mV. In current-clamp recordings, the resting membrane potential was kept at −70 mV by passing a holding current. Criteria to include cells in the analysis were visual confirmation of GFP in the pipette tip and absolute leak current <50 pA at −60 mV. Series resistance was typically <25 megohms, and experiments were discarded if >45 megohms. In all experiments, control or Sox11 overexpression treatments were blind to the operator.

### snRNA-seq processing

We used STARsolo 2.7 to align the snRNA-seq reads to the GRCm38 mouse genome. Multi-mapped reads were excluded, and both exonic and intronic counts were considered. We used default parameters to count unique molecular identifier and filter high-quality cells to generate gene-by-cell count matrices. Quality control (QC) and downstream data processing were performed using scran and scDblFinder bioconductor libraries (*77*, *78*). All scripts used to perform the present analysis were included in the companion website https://github.com/chernolabs/NeuronalSwitch.

For dataset 1, we removed 80 nuclei with <1000 detected features and 636 nuclei identified as doublets by scDblFinder out of the 17,046 nuclei that passed the STARsolo quality filter. We also eliminated 839 low-quality nuclei identified by either (i) a multivariate outlier detection procedure (adjOutlyingness function of robustbase R library) based on low library sizes, low number of detected features, and large mitochondrial (Mt) content, or (ii) presenting more than 1% of Mt counts. Features with >20 molecules and detected in >1 and < 80% of filtered nuclei were retained. In addition, we removed specific genes (*Ehd2*, *Espl1*, *Jarid1d*, *Pnpla4*, *Rps4y1*, *Xist*, *Tsix*, *Eif2s3y*, *Ddx3y*, *Uty*, *Kdm5d*, *Rpl26*, *Gstp1*, *Rpl35a*, *Erh*, *Slc25a5*, *Pgk1*, *Eno1*, *Tubb2a*, *Emc4*, and *Scg5*) to mitigate possible sex and stress effects on downstream analysis (*28*). The data were normalized using the scran library in accordance with the OSCA pipeline (*79*). We executed a quick clustering for each time point independently, normalized nuclei in each cluster separately, and rescaled the size factors to be comparable across clusters. Last, we computed the log_2_ of the data and considered a pseudo-count value of 1 to generate a normalized expression matrix. After scaling and log-normalizing gene expression values, we used the scran modelGeneVar function to identify the 3000 most variable genes in their log-expression profiles.

For dataset 2, 32,621 nuclei successfully passed the STARsolo quality filter. Subsequently, we identified and discarded 1015 doublets and 4808 low-quality nuclei using the QC protocol applied to dataset 1. The entire curation process yielded a high-quality dataset comprising 26,798 nuclei and 14,220 informative features. We considered inter-nucleus similarities using the Pearson correlation measure within the subspace spanned by the top 20 principal components. We lastly retained for further analysis 26,716 nuclei that were part of graph components larger than 30 nodes. We then used the batchelor Bioconductor package to investigate the biological reproducibility between datasets 1 and 2. Some dataset 1 nuclei occupied a region in reduced dimensionality spaces [principal components analysis (PCA), *t*-SNE, and uniform manifold approximation and projection (UMAP)] not represented in dataset 2. Using a *k* = 20 mKNN graph estimated from the largest 20 PCA components (similarities obtained from Pearson correlation values), we verified that these nuclei formed two Louvain clusters composed of 1071 nuclei from 4-week-old samples. Noticeably, we found that 85% of nuclei in these clusters exhibited higher expression of male-associated genes (*Ddx3y*, *Uty*, and *Eif2s3y*) than female-associated genes (*Xist* and *Tsix*), suggesting that they could belong to a single male mouse, potentially a biological outlier. Therefore, we reprocessed de novo dataset 1, excluding these nuclei that resulted in 14,441 high-quality nuclei and 13,353 features. To get a proxy of the biologically relevant manifold, we constructed an mKNN graph (*k* = 40) considering the Pearson correlation similarity measure estimated from the 20 largest PCA components. As we did with dataset 2, we discarded nuclei that were found in small components (<30 elements) of this mKNN graph. The remaining 14,367 nuclei were grouped into communities considering the Louvain algorithm. This initial graph-based clustering was further refined to produce 13 communities for which specific markers were identified (see Results). We used Seurat’s label transfer functionality to impute cluster labels for dataset 2. Only seven nuclei were identified as NB2, probably due to the fact that dataset 2 did not include a time point at 1 week. These nuclei were disregarded for subsequent analysis. We determined DEGs between nuclei groups using the FindMarkers function from the scran library (pairwise *t* test). Cluster marker genes were identified as highly ranked DEGs between the query group and all other clusters.

### Pseudo-time determination

The slingshot 2.4.0 R package was used to fit developmental trajectories to nuclei of the following clusters: RGL, NPC, NB1, NB2, GCimm1, GCimm2, GCyoung, and GCmat1 (*80*). We excluded from this analysis GCmat2 nuclei and 383 GCmat1 nuclei with strong expression signal of ventral genes (*81*, *82*). We considered the UMAP (three-dimensional) low-dimensional coordinates to fit principal curves and produce pseudo-time estimations (PCA-3dim and PCA-20dim produced similar density profiles along the corresponding pseudo-time coordinates). A similar procedure was used for dataset 2. For the sake of the pseudo-time analysis, a cluster refinement of the Seurat label transferred partition was considered to correct visible misclassifications observed in UMAP space. This issue affected <3% of nuclei (mainly involving astrocytes, pericytes, RGL, and NPC nuclei; see 05_DS2_SeuratLT.R in the web companion site).

### TF expression cascades

We considered 703 mouse TFs listed in the dorothea 1.8.0 R package that passed the QC feature filtering step (*83*). We tested whether these genes were significantly expressed along the pseudo-time coordinate using the difftest function of the TSCAN 1.34 R package. Likelihood ratio tests were performed to compare a generalized additive model with a constant fit to get the *P* values, with 201 TFs showing false discovery rate (FDR)–adjusted *q* value < 0.05. A custom script (09_cascades_Tfs.R in the web companion site) was used to identify TFs that were shutdown (112) or turned on (69) at specific clusters in this dataset (Fig. 3C and fig. S4C).

### Gene Ontology analysis

GO enrichment analyses was performed using ShinyGO 0.77 (http://bioinformatics.sdstate.edu/go/) (*84*).

### SCENIC regulons

SCENIC 1.3.1 R package was used on dataset 2 to uncover relevant TFs and their targets [regulons; (*85*)] along the developmental path (Fig. 3B and fig. S4B). RSSs (*50*) were used to analyze changes in regulon activity between consecutive states. Groups of nuclei were defined using a pseudo-time window of 5 U centered around local maximums (peaks) of the pseudo-time density distribution. This procedure resulted in 3840, 799, 7270, and 4119 nuclei assigned to the first, second, third, and fourth peaks, respectively.

### Visualization and graphical reports

We used Force Atlas 2 layout algorithm, implemented in Gephi to produce 2-dim visualization of mKNN graphs (*86*). Heatmaps, volcano plots, violin plots, dot plots, and spike plots were produced using the scX R package (https://chernolabs.github.io/scX/) (*88*).

### Statistical analysis

All data were presented as means ± SEM, and analyses were performed blind to the operator. Electrophysiological data were analyzed using Clampfit, immunofluorescence colocalization was verified using Zen blue 3.2, and RNAscope images were quantified using ImageJ software. Analyses were performed using GraphPad Prism 10 (GraphPad Software, CA, USA). Two-group comparisons were conducted with unpaired Student’s *t* test after data passed three normality tests (D’Agostino and Pearson, Shapiro-Wilk, and Kolmogorov-smirnov) or Mann-Whitney *U* test for nonparametric data. Comparisons across multiple groups were done using Kruskal-Wallis test, and data shown in Fig. 7G was compared using two-way analysis of variance (ANOVA).

*Note added in proof*: After acceptance, the authors became aware of a recently published paper: T. V. Waichman, M. L. Vercesi, A. A. Berardino, M. S. Beckel, D. Giacomini, N. B. Rasetto, M. Herrero, D. J. Di Bella, P. Arlotta, A. F. Schinder, A. Chernomoretz, scX: a user-friendly tool for scRNAseq exploration. *Bioinform. Adv.* **4**, vbae062 (2024). This added reference describes the routines that have been used to interrogate the dataset and make figures.
