# Supplementary material for: Transcriptional dynamics orchestrating the development and integration of neurons born in the adult hippocampus
Source: Sci Adv. 2024 Jul 19;10(29):eadp6039. doi: 10.1126/sciadv.adp6039 (PMC11259177; doi:10.1126/sciadv.adp6039)
Supplement: Supplementary file 1 — Figs. S1 to S9 Legends for tables S1 to S5 [file sciadv.adp6039_sm.pdf]

Science Advances
AAAS

Supplementary Materials for

Transcriptional dynamics orchestrating the development and integration of neurons born in the adult hippocampus

Natalí B. Rasetto *et al.*

Corresponding author: Alejandro F. Schinder, aschinder@leloir.org.ar; Ariel Chernomoretz, achernomoretz@leloir.org.ar; Paola Arlotta, paola_arlotta@harvard.edu

Sci. Adv. **10**, eadp6039 (2024)
DOI: 10.1126/sciadv.adp6039

The PDF file includes:

Figs. S1 to S9
Legends for tables S1 to S5

Other Supplementary Material for this manuscript includes the following:

Tables S1 to S5

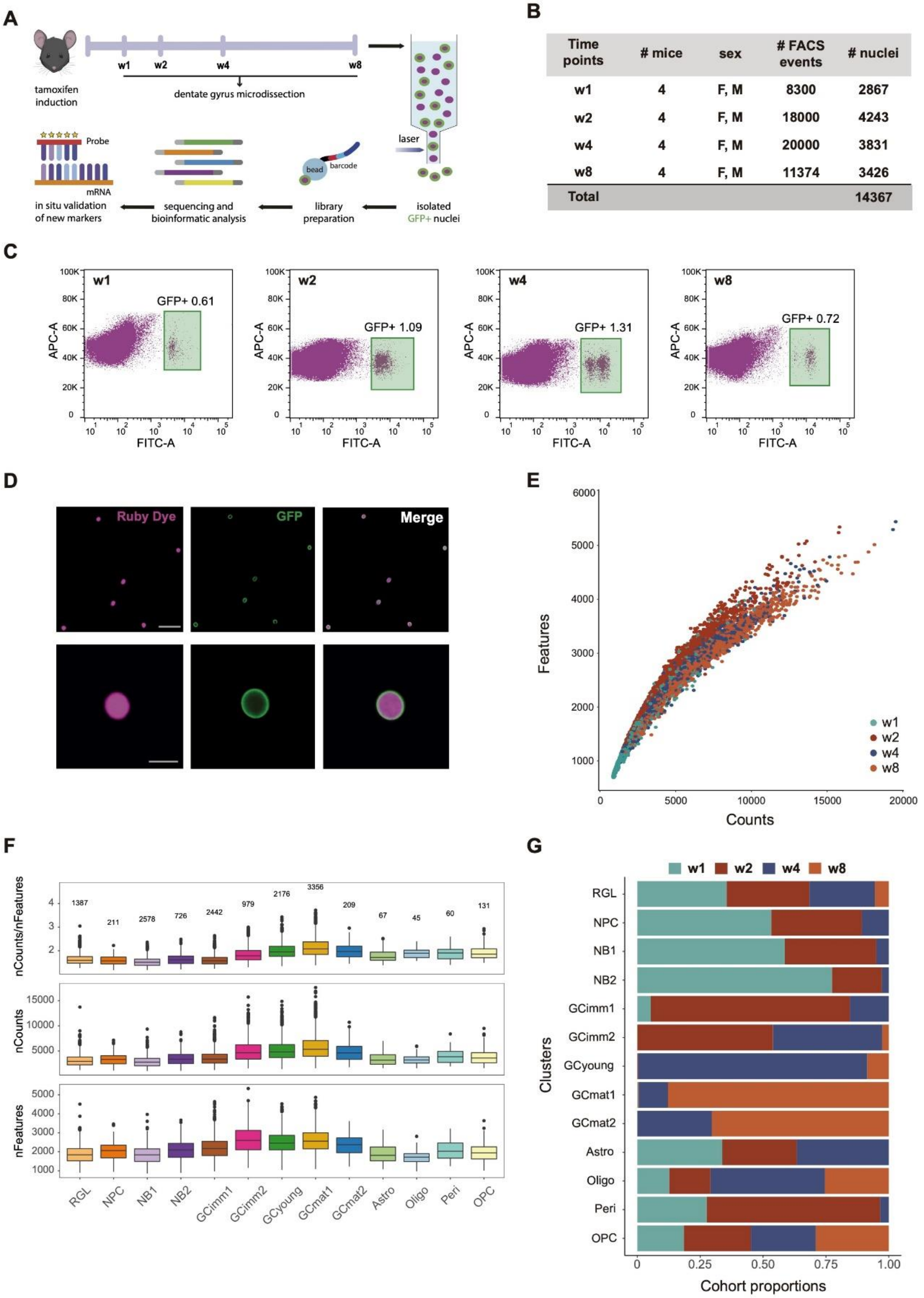

B

Time points	# mice	sex	# FACS events	# nuclei
w1	4	F, M	8300	2867
w2	4	F, M	18000	4243
w4	4	F, M	20000	3831
w8	4	F, M	11374	3426
Total				14367

Figure S1

Figure S1. Experimental pipeline and quality control for dataset 1. (A) *Ascl1*^{CreERT2};*CAG*$^{floxStop\text{-}Sun1/sfGFP}$ mice received tamoxifen (TAM) injections to label nuclei from the progeny of RGLs and NPCs. Dentate giri were microdissected at the indicated timepoints (cohorts). Each cohort of GFP^{+} nuclei was FACS-purified and processed separately using 10x Chromium technology for cDNA library preparation and sequencing. Markers were studied by fluorescence *in situ* hybridization. **(B)** Nuclei composition for each cohort, indicating mice number and sex (F, females; M, males), number of FACS-sorted, and analyzed nuclei. **(C)** FACS sorting of Ruby^{+}/GFP^{+} nuclei. Scatter plots of ruby dye intensity vs. log(GFP intensity) for purified nuclei isolated at w1, w2, w4 and w8. Green squares highlight the GFP^{+}/Ruby^{+} population, with the number indicating % GFP^{+} nuclei in the total population. **(D)** Confocal images of FACS-purified Ruby^{+} nuclei exhibiting GFP anchored to the nuclear membrane. Scale bars: 50 µm (upper panels) and 10 µm (lower panels). **(E)** Quality measurements of snRNA-seq libraries. Scatter plot depicting the number of genes (features) vs. the number of mRNA molecules (counts) per nuclei for each timepoint. snRNA-seq detected similar number of genes and transcript molecules across cohorts. **(F)** Quality measurements of snRNA-seq for each cluster. Box plots depict the number of counts, features, and their ratio. snRNA-seq detected similar number of genes and transcript molecules across clusters**. (G)** Contribution of timed cohorts to cluster composition.

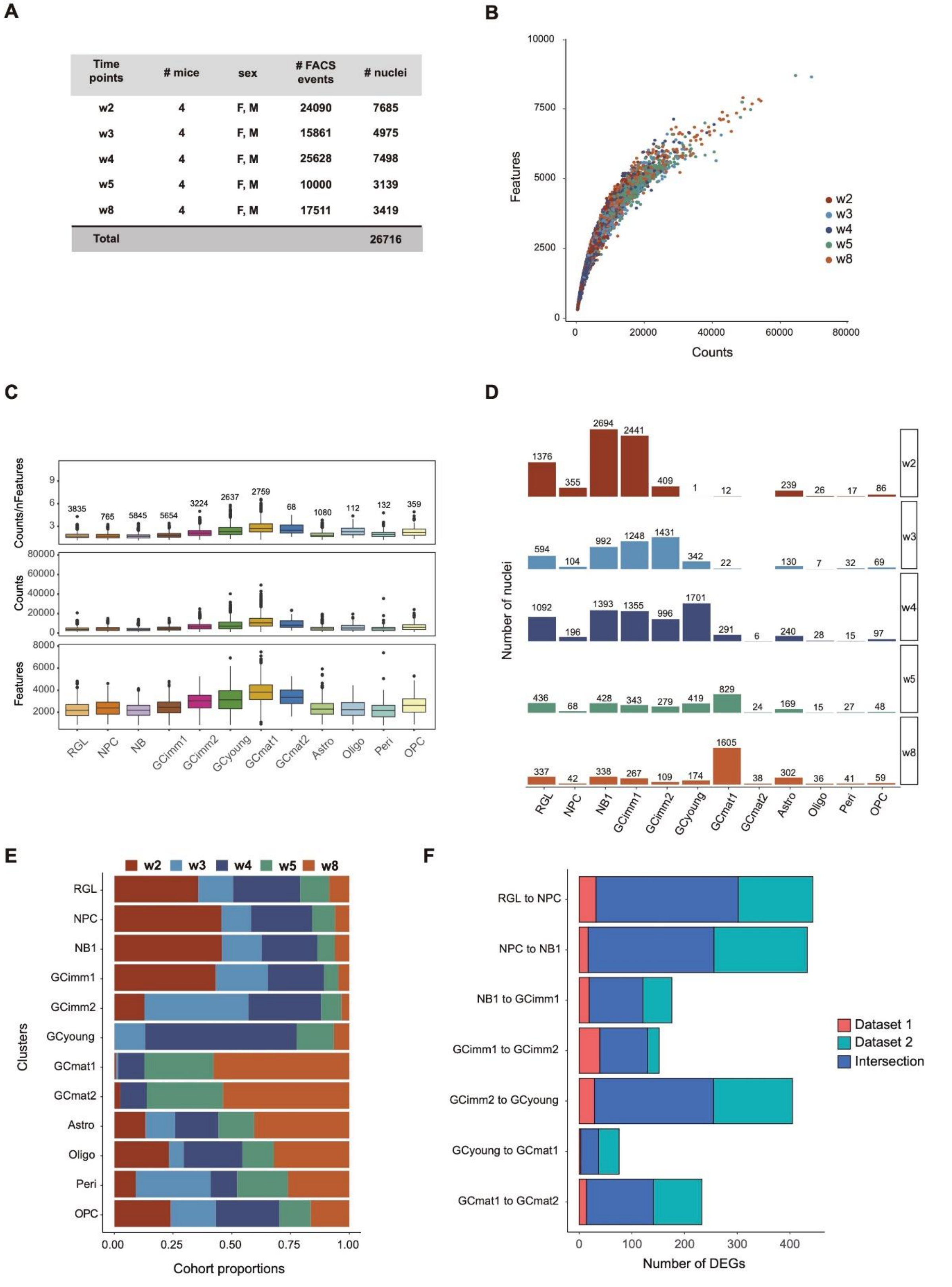

A

Time points	# mice	sex	# FACS events	# nuclei
w2	4	F, M	24090	7685
w3	4	F, M	15861	4975
w4	4	F, M	25628	7498
w5	4	F, M	10000	3139
w8	4	F, M	17511	3419
Total				26716

Figure S2

Figure S2. Quality control and cluster distribution for dataset 2. (A) Nuclei composition for each cohort, indicating mice number and sex (F, females; M, males), number of FACS-sorted, and analyzed nuclei. **(B)** Quality measurements of snRNA-seq libraries. Scatter plot depicting the number of genes (Features) vs. the number of mRNA molecules (counts) per nuclei for each timepoint. snRNA-seq detected similar number of genes and transcript molecules across cohorts. **(C)** Quality measurements of snRNA-seq for each cluster. Box plots depict the number of counts, features, and their ratio. snRNA-seq detected similar number of genes and transcript molecules across clusters**. (D)** Nuclei distribution in all clusters for each neuronal cohort. **(E)** Contribution of timed cohorts to cluster composition. **(F)** Bar chart indicating the number of DEGs between adjacent cluster transitions unique for dataset 1 (pink), dataset 2 (turquoise) and their intersection (blue).

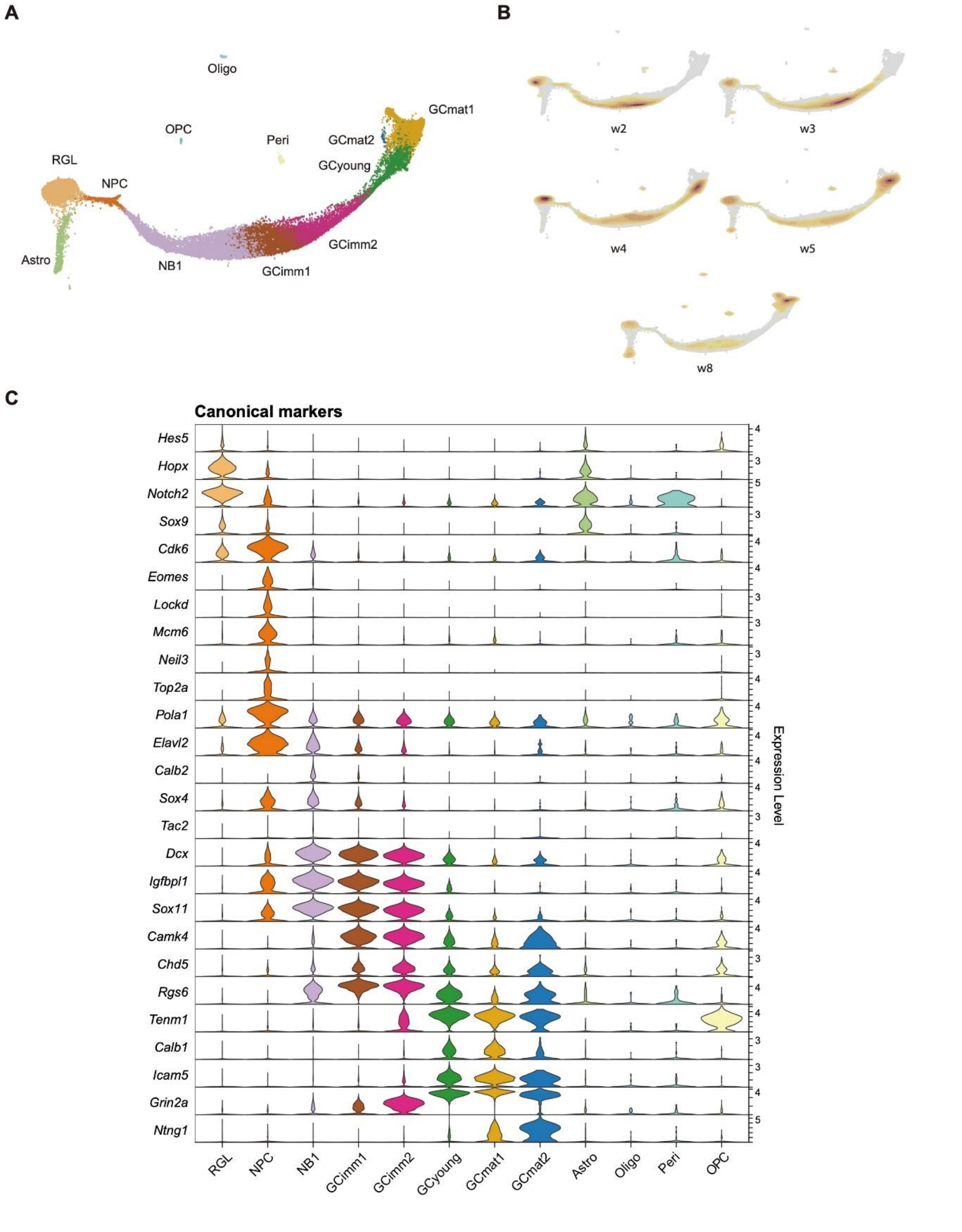
A
Oligo
OPC
Peri
GCmat1
GCmat2
GCyoung
RGL
NPC
Astro
NB1
GCimm1
GCimm2
B
w2
w3
w4
w5
w8
C
Canonical markers
Hes5
Hopx
Notch2
Sox9
Cdk6
Eomes
Lockd
Mcm6
Neil3
Top2a
Pola1
Elavl2
Calb2
Sox4
Tac2
Dcx
Igfbpl1
Sox11
Camk4
Chd5
Rgs6
Tenm1
Calb1
Icam5
Grin2a
Ntng1
Expression Level
RGL
NPC
NB1
GCimm1
GCimm2
GCyoung
GCmat1
GCmat2
Astro
Oligo
Peri
OPC

Figure S3

Figure S3. Cluster identity and transitions in dataset 2. (A) mKNN graph displaying cluster identity. **(B)** Progression of each cohort and their localization over the mKNN graph. Nuclei density is indicated by the yellow (low) to red (high) gradient. **(C)** Violin plot showing the expression level of canonical marker genes for the defined clusters. Note that most identified clusters are conserved with dataset 1, highlighting the reproducibility between experiments.

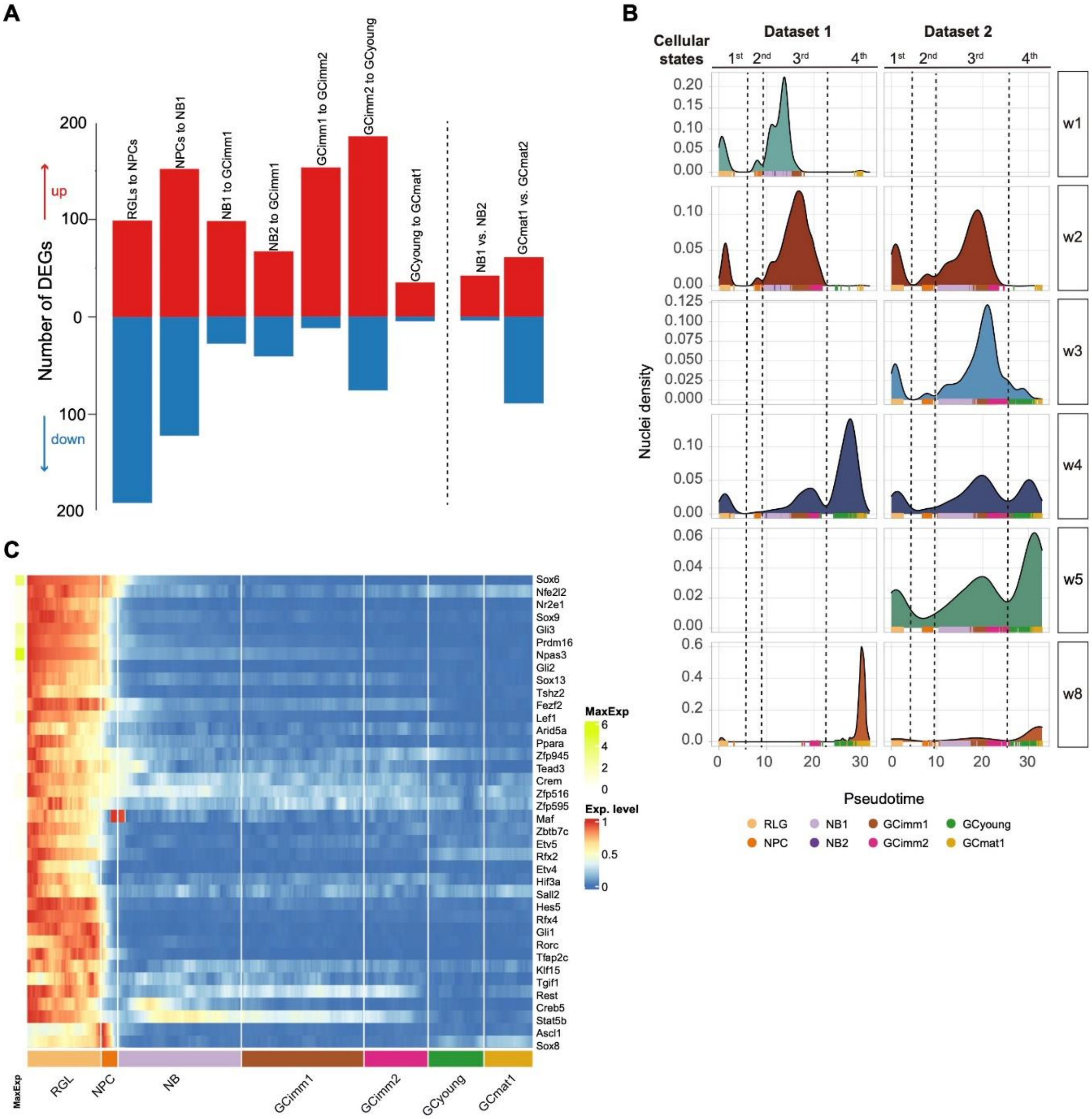
A
Number of DEGs
up
down
RGLs to NPCs
NPCs to NB1
NB1 to GCimm1
NB2 to GCimm1
GCimm1 to GCimm2
GCimm2 to GCyoung
GCyoung to GCmat1
NB1 vs. NB2
GCmat1 vs. GCmat2
B
Cellular states
Dataset 1
Dataset 2
Nuclei density
Pseudotime
w1
w2
w3
w4
w5
w8
RLG
NPC
NB1
NB2
GCimm1
GCimm2
GCyoung
GCmat1
C
MaxExp
Exp. level
RGL
NPC
NB
GCimm1
GCimm2
GCyoung
GCmat1
Sox6
Nfe2l2
Nr2e1
Sox9
Gli3
Prdm16
Npas3
Gli2
Sox13
Tshz2
Fezf2
Lef1
Arid5a
Ppara
Zfp945
Tead3
Crem
Zfp516
Zfp595
Maf
Zbtb7c
Etv5
Rfx2
Etv4
Hif3a
Sall2
Hes5
Rfx4
Gli1
Rorc
Tfap2c
Klf15
Tgif1
Rest
Creb5
Stat5b
Ascl1
Sox8

Figure S4

Figure S4. Pseudotime and density profiles are conserved between datasets. (A) DEGs between adjacent cluster transitions indicated on top (dataset 1; DEGs listed in Table S3). Additional comparisons (non-adjacent) are shown on the right. Up- and downregulated genes are shown in red and blue. **(B)** Density distribution of all nuclei along the pseudotime progression for each cohort, comparing datasets 1 and 2. The four cellular states are conserved: 1st RGL; 2nd NPC; 3rd NB1-GCimm1-GCimm2; 4th GCyoung-GCmat1. Dashed lines depict major transitions along development. Color codes below denote cluster identity **(C)** Heatmap displaying the row-wise normalized expression of transcription factors specific for RGLs, depicting additional transcripts to those shown in Fig. 3C. Maximum expression (MaxExp, colored scale on the right) is shown on the left. Pseudocolor scale on the right denotes mean expression level. Color-coded clusters are indicated below.

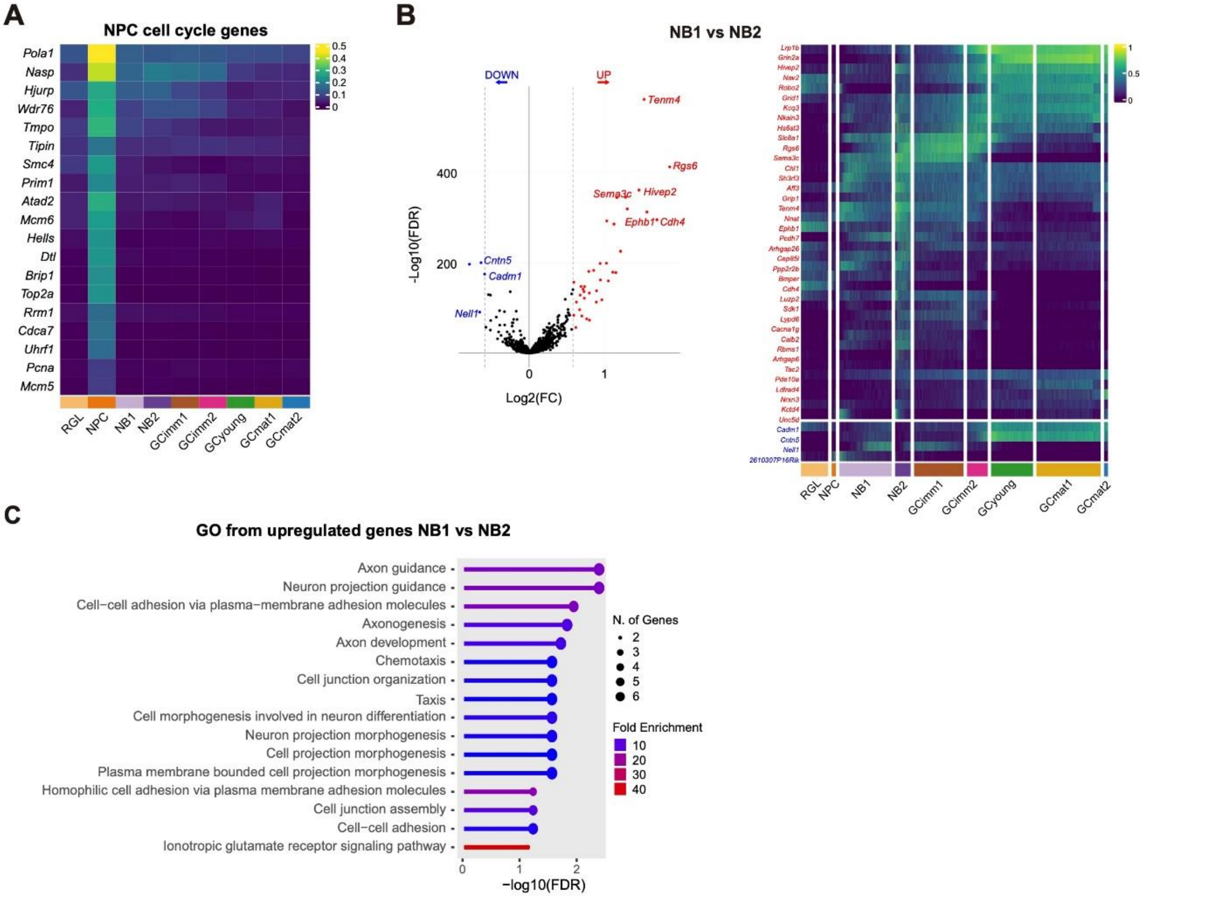
A
NPC cell cycle genes
Pola1
Nasp
Hjurp
Wdr76
Tmpo
Tipin
Smc4
Prim1
Atad2
Mcm6
Hells
Dtl
Brip1
Top2a
Rrm1
Cdca7
Uhrf1
Pcna
Mcm5
0.5
0.4
0.3
0.2
0.1
0
RGL
NPC
NB1
NB2
GCimm1
GCimm2
GCyoung
GCmat1
GCmat2
B
NB1 vs NB2
DOWN
UP
Tenm4
Rgs6
Sema3c
Hivep2
Ephb1
Cdh4
Cntn5
Cadm1
Nell1
400
200
-Log10(FDR)
0
1
Log2(FC)
1
0.5
0
RGL
NPC
NB1
NB2
GCimm1
GCimm2
GCyoung
GCmat1
GCmat2
C
GO from upregulated genes NB1 vs NB2
Axon guidance
Neuron projection guidance
Cell−cell adhesion via plasma−membrane adhesion molecules
Axonogenesis
Axon development
Chemotaxis
Cell junction organization
Taxis
Cell morphogenesis involved in neuron differentiation
Neuron projection morphogenesis
Cell projection morphogenesis
Plasma membrane bounded cell projection morphogenesis
Homophilic cell adhesion via plasma membrane adhesion molecules
Cell junction assembly
Cell−cell adhesion
Ionotropic glutamate receptor signaling pathway
0
1
2
−log10(FDR)
N. of Genes
2
3
4
5
6
Fold Enrichment
10
20
30
40

Figure S5

Figure S5. Molecular signatures of early stages of adult neurogenesis. (A) Heatmap showing the mean of row-wise normalized expression of canonical cell-cycle markers across clusters. Pseudocolor scale on the right denotes mean expression level. **(B)** Comparison between NB1 and NB2. Left panel: volcano plot showing differential expression analysis (FC $\geq$ 1.5 or $\leq$ -1.5 and FDR $\leq$ 0.05) with relevant gene examples. Right panel: heatmap showing the row-wise normalized expression of up- (red) and downregulated DEGs (blue) across clusters (top 50 genes per transition). Pseudocolor scale on the right denotes mean expression level. DEGs are listed in Table S3. **(C)** Top GO biological processes for the enrichment analysis of DEGs in the transition from NB1 to NB2. FDR cutoff = 0.05 (using ShinyGO 0.77). All data in the figure correspond to dataset 1.

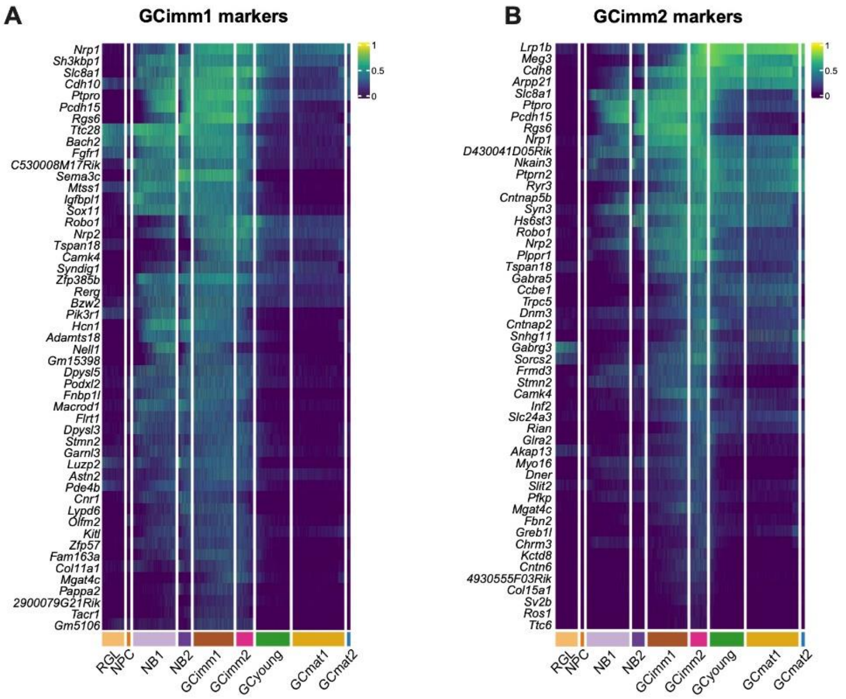
A
GCimm1 markers
Nrp1
Sh3kbp1
Slc8a1
Cdh10
Ptpro
Pcdh15
Rgs6
Ttc28
Bach2
Fgfr1
C530008M17Rik
Sema3c
Mtss1
Igfbpl1
Sox11
Robo1
Nrp2
Tspan18
Camk4
Syndig1
Zfp385b
Rerg
Bzw2
Pik3r1
Hcn1
Adamts18
Nell1
Gm15398
Dpysl5
Podxl2
Fnbp1l
Macrod1
Flrt1
Dpysl3
Stmn2
Garnl3
Luzp2
Astn2
Pde4b
Cnr1
Lypd6
Olfm2
Kitl
Zfp57
Fam163a
Col11a1
Mgat4c
Pappa2
2900079G21Rik
Tacr1
Gm5106
1
0.5
0
RGL
NPC
NB1
NB2
GCimm1
GCimm2
GCyoung
GCmat1
GCmat2
B
GCimm2 markers
Lrp1b
Meg3
Cdh8
Arpp21
Slc8a1
Ptpro
Pcdh15
Rgs6
Nrp1
D430041D05Rik
Nkain3
Ptprn2
Ryr3
Cntnap5b
Syn3
Hs6st3
Robo1
Nrp2
Plppr1
Tspan18
Gabra5
Ccbe1
Trpc5
Dnm3
Cntnap2
Snhg11
Gabrg3
Sorcs2
Frmd3
Stmn2
Camk4
Inf2
Slc24a3
Rian
Glra2
Akap13
Myo16
Dner
Slit2
Pfkp
Mgat4c
Fbn2
Greb1l
Chrm3
Kctd8
Cntn6
4930555F03Rik
Col15a1
Sv2b
Ros1
Ttc6
1
0.5
0
RGL
NPC
NB1
NB2
GCimm1
GCimm2
GCyoung
GCmat1
GCmat2

Figure S6

Figure S6. Marker genes for GCimm. (A,B) Heatmaps showing the row-wise normalized expression of marker genes for GCimm1 and GCimm2 across clusters (top 50 genes). Marker genes were selected as described in the Methods section. Pseudocolor scales on the right denote mean expression level. Data correspond to dataset 1.

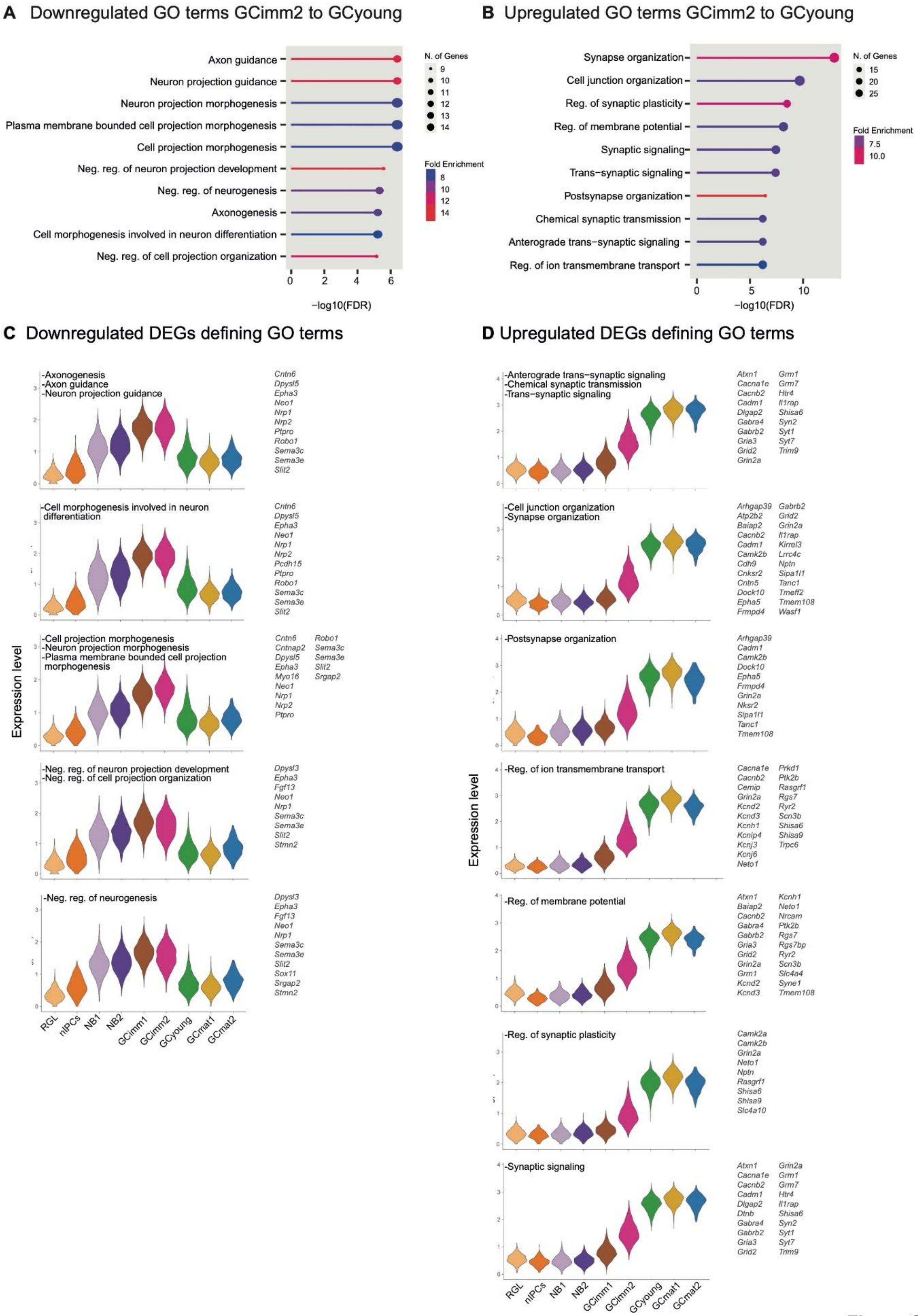
A Downregulated GO terms GCimm2 to GCyoung
Axon guidance
Neuron projection guidance
Neuron projection morphogenesis
Plasma membrane bounded cell projection morphogenesis
Cell projection morphogenesis
Neg. reg. of neuron projection development
Neg. reg. of neurogenesis
Axonogenesis
Cell morphogenesis involved in neuron differentiation
Neg. reg. of cell projection organization
−log10(FDR)
N. of Genes
Fold Enrichment
B Upregulated GO terms GCimm2 to GCyoung
Synapse organization
Cell junction organization
Reg. of synaptic plasticity
Reg. of membrane potential
Synaptic signaling
Trans−synaptic signaling
Postsynapse organization
Chemical synaptic transmission
Anterograde trans−synaptic signaling
Reg. of ion transmembrane transport
−log10(FDR)
N. of Genes
Fold Enrichment
C Downregulated DEGs defining GO terms
-Axonogenesis
-Axon guidance
-Neuron projection guidance
Cntn6 Dpysl5 Epha3 Neo1 Nrp1 Nrp2 Ptpro Robo1 Sema3c Sema3e Slit2
-Cell morphogenesis involved in neuron differentiation
Cntn6 Dpysl5 Epha3 Neo1 Nrp1 Nrp2 Pcdh15 Ptpro Robo1 Sema3c Sema3e Slit2
-Cell projection morphogenesis
-Neuron projection morphogenesis
-Plasma membrane bounded cell projection morphogenesis
Cntn6 Cntnap2 Dpysl5 Epha3 Myo16 Neo1 Nrp1 Nrp2 Ptpro Robo1 Sema3c Sema3e Slit2 Srgap2
-Neg. reg. of neuron projection development
-Neg. reg. of cell projection organization
Dpysl3 Epha3 Fgf13 Neo1 Nrp1 Sema3c Sema3e Slit2 Stmn2
-Neg. reg. of neurogenesis
Dpysl3 Epha3 Fgf13 Neo1 Nrp1 Sema3c Sema3e Slit2 Sox11 Srgap2 Stmn2
Expression level
RGL nIPCs NB1 NB2 GCimm1 GCimm2 GCyoung GCmat1 GCmat2
D Upregulated DEGs defining GO terms
-Anterograde trans−synaptic signaling
-Chemical synaptic transmission
-Trans−synaptic signaling
Atxn1 Cacna1e Cacnb2 Cadm1 Dlgap2 Gabra4 Gabrb2 Gria3 Grid2 Grin2a Grm1 Grm7 Htr4 Il1rap Shisa6 Syn2 Syt1 Syt7 Trim9
-Cell junction organization
-Synapse organization
Arhgap39 Atp2b2 Baiap2 Cacnb2 Cadm1 Camk2b Cdh9 Cnksr2 Cntn5 Dock10 Epha5 Frmpd4 Gabrb2 Grid2 Grin2a Il1rap Kirrel3 Lrrc4c Nptn Sipa1l1 Tanc1 Tmeff2 Tmem108 Wasf1
-Postsynapse organization
Arhgap39 Cadm1 Camk2b Dock10 Epha5 Frmpd4 Grin2a Nksr2 Sipa1l1 Tanc1 Tmem108
-Reg. of ion transmembrane transport
Cacna1e Cacnb2 Cemip Grin2a Kcnd2 Kcnd3 Kcnh1 Kcnip4 Kcnj3 Kcnj6 Neto1 Prkd1 Ptk2b Rasgrf1 Rgs7 Ryr2 Scn3b Shisa6 Shisa9 Trpc6
-Reg. of membrane potential
Atxn1 Baiap2 Cacnb2 Gabra4 Gabrb2 Gria3 Grid2 Grin2a Grm1 Kcnd2 Kcnd3 Kcnh1 Neto1 Nrcam Ptk2b Rgs7 Rgs7bp Ryr2 Scn3b Slc4a4 Syne1 Tmem108
-Reg. of synaptic plasticity
Camk2a Camk2b Grin2a Neto1 Nptn Rasgrf1 Shisa6 Shisa9 Slc4a10
-Synaptic signaling
Atxn1 Cacna1e Cacnb2 Cadm1 Dlgap2 Dtnb Gabra4 Gabrb2 Gria3 Grid2 Grin2a Grm1 Grm7 Htr4 Il1rap Shisa6 Syn2 Syt1 Syt7 Trim9
Expression level
RGL nIPCs NB1 NB2 GCimm1 GCimm2 GCyoung GCmat1 GCmat2

Figure S7

Figure S7. Switch in biological processes during the exit from the immature state. (A, B) GO biological processes are shown for the enrichment analysis of the top-50 DEGs in the transition from GCimm2 to GCyoung. FDR cutoff = 0.05 (using ShinyGO 0.77). **(C, D)** Violin plots showing the mean expression levels of DEGs for downregulated **(C)** and upregulated GO terms **(D)** for the defined clusters. All data in the figure correspond to dataset 1. DEGs are listed in Table S3.

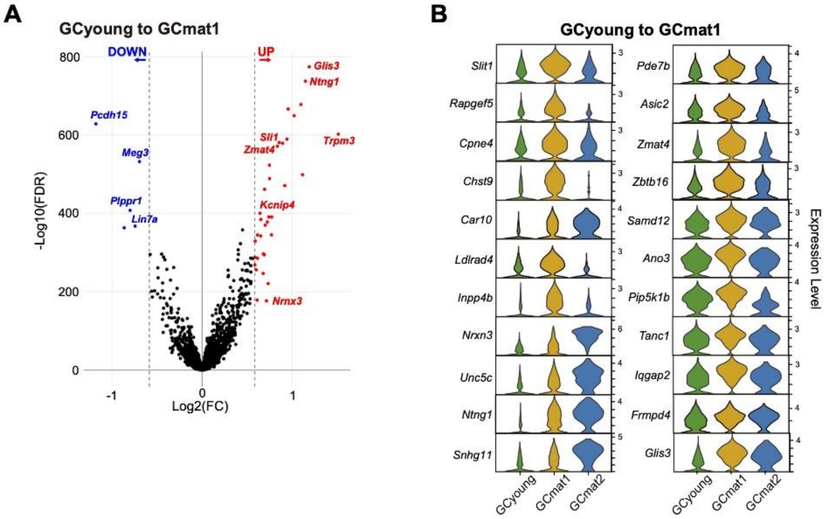

A
GCyoung to GCmat1
DOWN
UP
Glis3
Ntng1
Pcdh15
Trpm3
Slit1
Zmat4
Meg3
Plppr1
Lin7a
Kcnip4
Nrxn3
-Log10(FDR)
Log2(FC)
B
GCyoung to GCmat1
Slit1
Rapgef5
Cpne4
Chst9
Car10
Ldlrad4
Inpp4b
Nrxn3
Unc5c
Ntng1
Snhg11
Pde7b
Asic2
Zmat4
Zbtb16
Samd12
Ano3
Pip5k1b
Tanc1
Iqgap2
Frmpd4
Glis3
Expression Level
GCyoung
GCmat1
GCmat2

Figure S8

Figure S8. GCyoung and GCmat1 displays subtle transcriptomic differences and share nuclei with ventral signature. (A) Volcano plot showing differential expression analysis (FC ≥ 1.5 or ≤ -1.5 and FDR ≤ 0.05) displaying relevant gene examples. All DEGs are listed in Table S3. **(B)** Violin plots showing the expression level of upregulated DEGs of the GCyoung to GCmat1 transition. All data in the figure correspond to dataset 1.

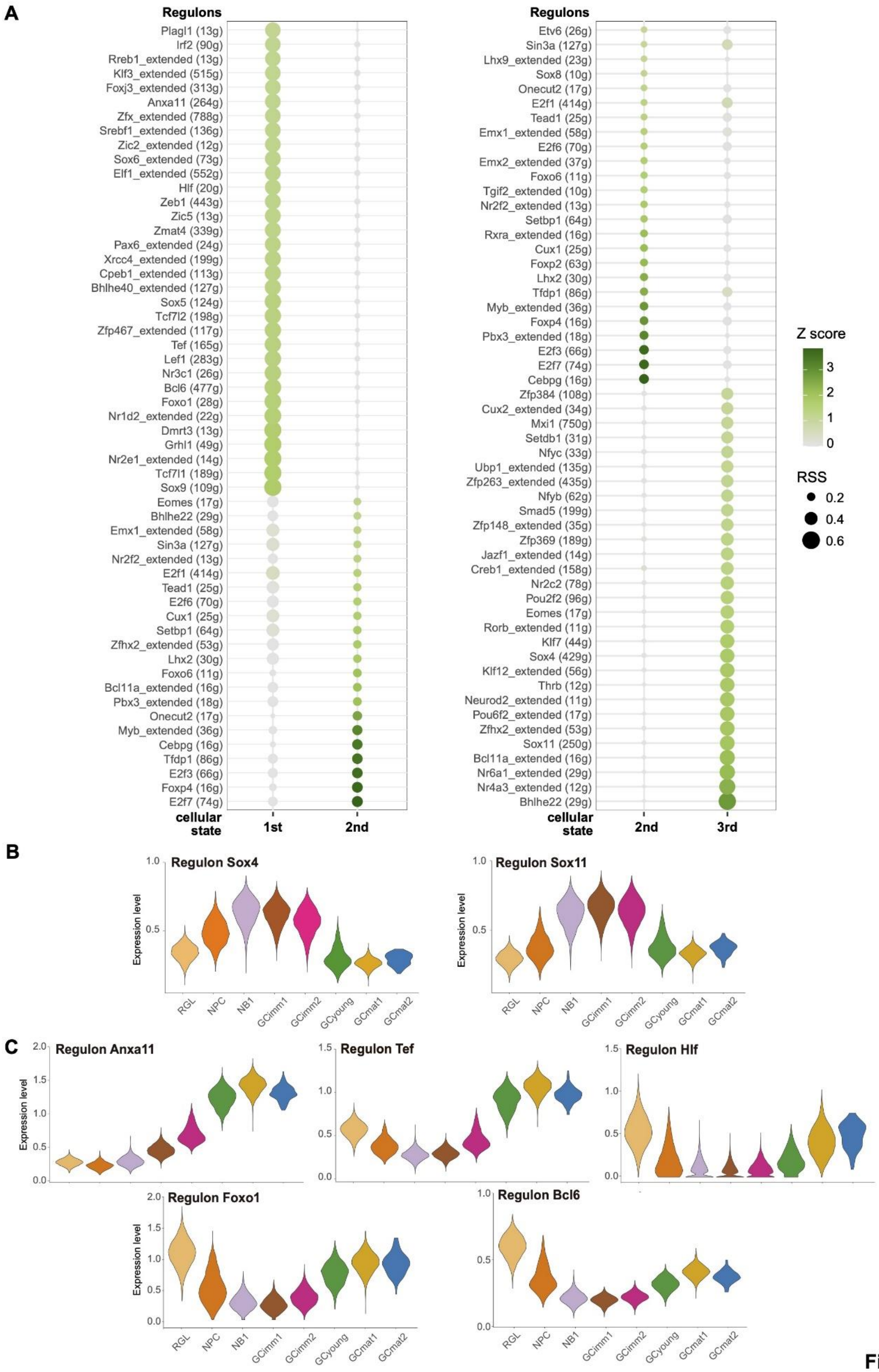
A
Regulons
Plagl1 (13g)
Irf2 (90g)
Rreb1_extended (13g)
Klf3_extended (515g)
Foxj3_extended (313g)
Anxa11 (264g)
Zfx_extended (788g)
Srebf1_extended (136g)
Zic2_extended (12g)
Sox6_extended (73g)
Elf1_extended (552g)
Hlf (20g)
Zeb1 (443g)
Zic5 (13g)
Zmat4 (339g)
Pax6_extended (24g)
Xrcc4_extended (199g)
Cpeb1_extended (113g)
Bhlhe40_extended (127g)
Sox5 (124g)
Tcf7l2 (198g)
Zfp467_extended (117g)
Tef (165g)
Lef1 (283g)
Nr3c1 (26g)
Bcl6 (477g)
Foxo1 (28g)
Nr1d2_extended (22g)
Dmrt3 (13g)
Grhl1 (49g)
Nr2e1_extended (14g)
Tcf7l1 (189g)
Sox9 (109g)
Eomes (17g)
Bhlhe22 (29g)
Emx1_extended (58g)
Sin3a (127g)
Nr2f2_extended (13g)
E2f1 (414g)
Tead1 (25g)
E2f6 (70g)
Cux1 (25g)
Setbp1 (64g)
Zfhx2_extended (53g)
Lhx2 (30g)
Foxo6 (11g)
Bcl11a_extended (16g)
Pbx3_extended (18g)
Onecut2 (17g)
Myb_extended (36g)
Cebpg (16g)
Tfdp1 (86g)
E2f3 (66g)
Foxp4 (16g)
E2f7 (74g)
cellular state
1st
2nd
Regulons
Etv6 (26g)
Sin3a (127g)
Lhx9_extended (23g)
Sox8 (10g)
Onecut2 (17g)
E2f1 (414g)
Tead1 (25g)
Emx1_extended (58g)
E2f6 (70g)
Emx2_extended (37g)
Foxo6 (11g)
Tgif2_extended (10g)
Nr2f2_extended (13g)
Setbp1 (64g)
Rxra_extended (16g)
Cux1 (25g)
Foxp2 (63g)
Lhx2 (30g)
Tfdp1 (86g)
Myb_extended (36g)
Foxp4 (16g)
Pbx3_extended (18g)
E2f3 (66g)
E2f7 (74g)
Cebpg (16g)
Zfp384 (108g)
Cux2_extended (34g)
Mxi1 (750g)
Setdb1 (31g)
Nfyc (33g)
Ubp1_extended (135g)
Zfp263_extended (435g)
Nfyb (62g)
Smad5 (199g)
Zfp148_extended (35g)
Zfp369 (189g)
Jazf1_extended (14g)
Creb1_extended (158g)
Nr2c2 (78g)
Pou2f2 (96g)
Eomes (17g)
Rorb_extended (11g)
Klf7 (44g)
Sox4 (429g)
Klf12_extended (56g)
Thrb (12g)
Neurod2_extended (11g)
Pou6f2_extended (17g)
Zfhx2_extended (53g)
Sox11 (250g)
Bcl11a_extended (16g)
Nr6a1_extended (29g)
Nr4a3_extended (12g)
Bhlhe22 (29g)
cellular state
2nd
3rd
Z score
3
2
1
0
RSS
0.2
0.4
0.6
B
Regulon Sox4
Regulon Sox11
Expression level
1.0
0.5
RGL
NPC
NB1
GCimm1
GCimm2
GCyoung
GCmat1
GCmat2
C
Regulon Anxa11
Regulon Tef
Regulon Hlf
Regulon Foxo1
Regulon Bcl6
Expression level
2.0
1.5
1.0
0.5
0.0
RGL
NPC
NB1
GCimm1
GCimm2
GCyoung
GCmat1
GCmat2

Figure S9

Figure S9. Regulon expression for the different cellular states. (A) Regulon activity analysis based on the expression levels of TF targets comparing the 1st with the 2nd and 2nd to the 3rd states. Each regulon contains targets genes identified by SCENIC according to the conserved DNA binding motifs in regulatory regions. Regulon sizes are shown in parentheses. Extended regulons include targets inferred by binding motif similarity. Regulon compositions are listed in Table S5. RSS: Regulon specificity score [46]. The z score color scale depicts standardized expression activity values. **(B, C)** Violin plots displaying the mean expression of transcripts that compose the indicated regulons. All data in the figure correspond to dataset 2.

ADDITIONAL SUPPLEMENTAL MATERIAL (Excel files)

Table S1. DATASET 1. Top 100 genes with highest variability, corresponding to Figure 1E.

Table S2. DATASET 2. Differentially expressed genes for cluster transitions in dataset 2, corresponding to Figs. 3A; S2F.

Table S3. DATASET 1. Differentially expressed genes for cluster transitions in dataset 1, corresponding to Figs. S4A ; 4B,C; 5B,C; S2F, S5B, S8A.

Table S4. DATASET 1. Ion channels, neurotransmitter receptors, and molecules involved in synaptic transmission, corresponding to Figure 5E.

Table S5. DATASET 2. Regulons with differential expression between states, corresponding to Figures 8A, S9A.
